# Destabilization of β Cell FIT2 by saturated fatty acids alter lipid droplet numbers and contribute to ER stress and diabetes

**DOI:** 10.1073/pnas.2113074119

**Published:** 2022-03-07

**Authors:** Xiaofeng Zheng, Qing Wei Calvin Ho, Minni Chua, Olga Stelmashenko, Xin Yi Yeo, Sneha Muralidharan, Federico Torta, Elaine Guo Yan Chew, Michelle Mulan Lian, Jia Nee Foo, Sangyong Jung, Sunny Hei Wong, Nguan Soon Tan, Nanwei Tong, Guy A. Rutter, Markus R. Wenk, David L. Silver, Per-Olof Berggren, Yusuf Ali

**Affiliations:** ^a^Lee Kong Chian School of Medicine, Nanyang Technological University Singapore, S308232, Singapore;; ^b^Singapore Eye Research Institute, Singapore General Hospital, S168751, Singapore;; ^c^Department of Endocrinology and Metabolism, Center for Diabetes and Metabolism Research, West China Hospital, Sichuan University, Chengdu 610041, People’s Republic of China;; ^d^Institute of Molecular and Cell Biology, Agency for Science, Technology and Research, S138667, Singapore;; ^e^Department of Psychological Medicine, Yong Loo Lin School of Medicine, National University of Singapore, S119228, Singapore;; ^f^Singapore Lipidomics Incubator, Department of Medicine, National University of Singapore, S117456, Singapore;; ^g^Singapore Lipidomics Incubator, Department of Biochemistry, Life Sciences Institute and Yong Loo Lin School of Medicine, National University of Singapore, S117456, Singapore;; ^h^Human Genetics, A*STAR, Genome Institute of Singapore, S138672, Singapore;; ^i^Department of Physiology, Yong Loo Lin School of Medicine, National University of Singapore, S117593, Singapore;; ^j^School of Biological Sciences, Nanyang Technological University Singapore, S637551, Singapore;; ^k^Section of Cell Biology and Functional Genomics, Division of Diabetes, Endocrinology, and Metabolism, Department of Metabolism, Digestion, and Reproduction, Imperial College London, London SW7 2AZ, United Kingdom;; ^l^Le Centre de recherche du Centre hospitalier de l’Université de Montréal (CR-CHUM), University of Montréal, Montréal, QC H2X 0A9, Canada;; ^m^Signature Research Program in Cardiovascular and Metabolic Disorders, Duke–National University of Singapore Graduate Medical School, S169857, Singapore;; ^n^The Rolf Luft Research Center for Diabetes and Endocrinology, Karolinska Institutet, Karolinska University Hospital, SE-171 76 Stockholm, Sweden

**Keywords:** FIT2, lipid droplets, pancreatic β cells, ER stress, diet-induced diabetes

## Abstract

Western-type diets are linked to obesity and diabetes partly because of their high–saturated fatty acid (SFA) content. We found that SFAs, but not unsaturated fatty acids (USFAs), reduced lipid droplets (LDs) within pancreatic β cells. Mechanistically, SFAs, but not USFAs, reduced LD formation by inducing S-acylation and proteasomal, mediated degradation of fat storage–inducing transmembrane protein 2 (FIT2), an endoplasmic reticulum (ER) resident protein important for LD formation. Targeted ablation of FIT2 reduced β cell LD numbers, lowered β cell ATP levels, reduced Ca^2+^ signaling, dampened vesicle exocytosis, down-regulated β cell transcription factors, up-regulated unfolded protein response genes, and finally, exacerbated diet-induced diabetes in mice. Subsequent mass spectrometry studies revealed increased C16:0 ceramide accumulation in islets of diet-induced diabetes mice lacking β cell FIT2. Inhibition of ceramide synthases ameliorated the enhanced ER stress and improved insulin secretion. FIT2 was reduced in mouse diabetic islets, and separately, overexpression of FIT2 increased the number of intracellular LDs and rescued SFA-induced ER stress and apoptosis, thereby highlighting the protective role of FIT2 and LDs against β cell lipotoxicity.

## Significance

With obesity on the rise, there is a growing appreciation for intracellular lipid droplet (LD) regulation. Here, we show how saturated fatty acids (SFAs) reduce fat storage–inducing transmembrane protein 2 (FIT2)–facilitated, pancreatic β cell LD biogenesis, which in turn induces β cell dysfunction and death, leading to diabetes. This mechanism involves direct acylation of FIT2 cysteine residues, which then marks the FIT2 protein for endoplasmic reticulum (ER)–associated degradation. Loss of β cell FIT2 and LDs reduces insulin secretion, increases intracellular ceramides, stimulates ER stress, and exacerbates diet-induced diabetes in mice. While palmitate and stearate degrade FIT2, unsaturated fatty acids such as palmitoleate and oleate do not, results of which extend to nutrition and diabetes.

Numerous studies show that saturated fatty acids (SFAs), but not unsaturated fatty acids (USFAs), trigger endoplasmic reticulum (ER) stress, cell dysfunction, and apoptosis in a myriad of cell types (1–4), including the pancreatic β cell. β cell vulnerability to free fatty acids (i.e., lipotoxicity) reduces insulin provision and leads to diabetes (5). As with most eukaryotic cells, β cells possess the ability to generate lipid droplets (LDs) from the ER (6). Beyond mere storage containers for de novo–assembled neutral lipids and cholesterol esters (7–9), LDs were shown to act as hubs for innate immune defense, modulators of nuclear function, and sites for protein storage and trafficking (10–12). The number of LDs present in a cell can dictate its energy rate, inflammation state, resistance to pathogens, ER stress, and cell death. LD biogenesis is a complex process involving many different ER resident enzymes, including those that are involved in the synthesis of neutral lipids and cholesterol esters such as diacylglycerol transferases (13). However, LD budding from the ER is largely regulated by a conserved tripartite ER protein machinery comprising of seipins, perilipins, and fat storage–inducing transmembrane proteins (FITs) (14, 15). A modification in each of these proteins affect LD budding from the ER (13).

Despite considerable attention and the link to many diseases, the contribution of LDs to β cell dysfunction and diabetes is just beginning to emerge. LDs are present in β cells, with notable differences in numbers between rodent and human β cells and in increasing numbers as β cells age (16). Prompted by our underpinning observation of a significant change in LD number between murine β cells exposed to SFAs with those exposed to USFAs, we looked at how SFAs may directly influence LD numbers in β cells and, in turn, how this regulation impacts β cell function and whole-body glucose homeostasis. We uncovered a mechanism by which SFAs reduce LD numbers, which involves the ubiquitously expressed FIT2/FITM2, with subsequent impact on β cell function, survival, and overall glucose homeostasis. FIT2 belongs to a unique family of evolutionarily conserved ER resident, 6-transmembrane domain, protein with its only other member being muscle cell–restricted FIT1/FITM1 (17–21). FIT2, a critical protein for LD formation, has been shown to promote ER lipid coalescence and LD formation, a function that is likely linked to its catalytic lipid phosphatase activity (15, 18, 22, 23). More recently, FIT2 was implicated in ER protein dysregulation, ER structure maintenance, and the maladaptive ER response in both yeast and mammalian cells (23, 24). Despite this, the role and importance of FIT2 in β cell LD formation, β cell function, and survival, especially during lipotoxicity, remains unknown.

Herein, we confirmed the presence of FIT2 in β cells of mouse islets and show that palmitate induces proteasomal degradation of FIT2 through direct palmitoylation. This loss, mimicked in mice with β cell–specific deletion of FIT2 (βFIT2KO), resulted in reduced β cell LD numbers and diet-induced diabetes, with exacerbated glucose intolerance due to a reduced insulin secretory response. We also observed ceramide accumulation, a key driver of β cell ER stress, in islets from βFIT2KO mice, and inhibition of ceramide synthase improved insulin secretion in βFIT2KO islets. Rescue of FIT2 in MIN6 cells, through overexpression, partially restored LD biogenesis, reduced ER stress, and ameliorated SFA-mediated β cell apoptosis (i.e., lipotoxicity). Beyond providing a conceptual advance in our understanding of how diet impacts the β cell, this study highlights the restoration of LD formation as a potential way of preserving normal β cell function in obesity.

## Results

### Palmitate and Stearate Reduce β Cell FIT2 Protein Levels.

**Fig. 1.**
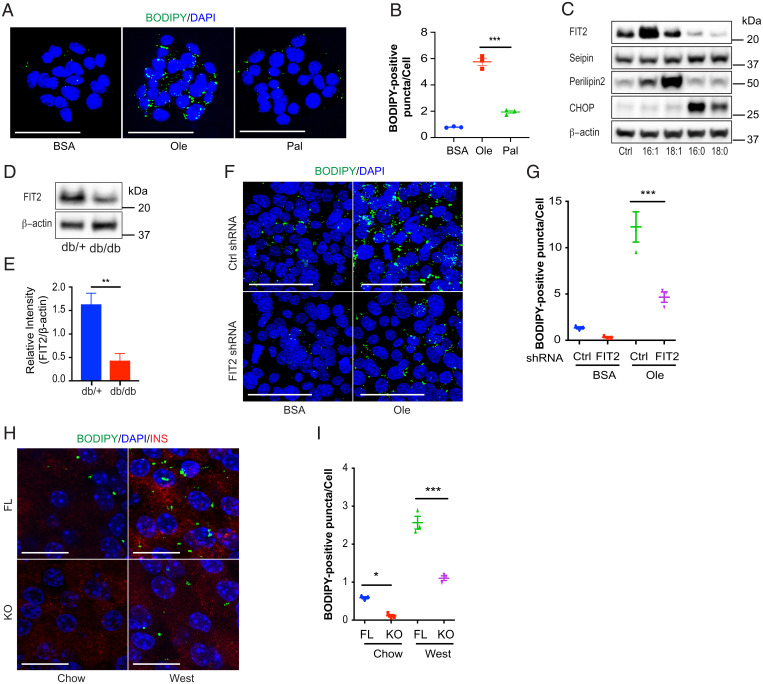
FA-mediated changes in β cell LD numbers relates to FIT2 protein abundance. (*A*) BODIPY staining of LDs in MIN6 cells treated with BSA, oleate, or palmitate (300 µM) for 24 h. LDs are stained with BODIPY (green) and nuclei stained with DAPI (blue). Images shown are maximum intensity projections. (Scale bar, 50 µm.) (*B*) Quantitation of BODIPY-positive puncta per cell using Image J. (>500 cells from three independent experiments were analyzed per group). (*C*) Representative immunoblot of several LD formation proteins after MIN6 cells were treated with 300 µM palmitoleate (16:1), oleate (18:1), palmitate (16:0), stearate (18:0), or BSA (control) for 24 h. (*D* and *E*) Representative immunoblot and semiquantitation of FIT2 protein in islets isolated from db/+ and db/db mice (16-wk-old male, *n* = 4). (*F*) BODIPY staining of LDs in control (Ctrl shRNA) or FIT2 knockdown (FIT2 shRNA) MIN6 cells treated with BSA or 300 µM oleate for 48 h. LDs were stained with BODIPY (green), and nuclei were stained with DAPI (blue). Images shown are maximum intensity projections. (Scale Bar, 50 µm.) (*G*) Quantitation of BODIPY-positive punctae per cell using Image J. BSA-treated control (Ctrl shRNA, blue) or FIT2 knockdown (FIT2 shRNA, red) and oleate-treated (300 µM) control (Ctrl shRNA, green) or FIT2 knockdown (FIT2 shRNA, purple) (>1,000 cells from three independent experiments were analyzed per group). (*H*) BODIPY staining of LDs in pancreas sections of chow-fed, floxed control (FL, blue) and βFIT2KO (KO, red) and western diet (West, 25 wk)–fed, floxed control (FL, green) and βFIT2KO (KO, purple). LDs were visualized using a BODIPY stain (green). β cells were stained with insulin (red) and nuclei stained with DAPI (blue). Images shown are maximum intensity projections. (Scale bar, 25 µm.) (*I*) Quantitation of BODIPY-positive puncta per cell using Image J (*n* = 3, ≥9 islets from three mice were analyzed per group). Values shown are mean ± SEM; *P < 0.05; **P < 0.01; and ***P < 0.001 (*B*: one-way ANOVA with Tukey’s post hoc test, *D*: two-tailed Student's *t* test, and *G* and *I*: two-way ANOVA with Šidák post hoc test).

To recapitulate β cell vulnerability to SFAs, we first exposed clonal MIN6 cells (25) to physiologically relevant levels of either oleate (300 µM) or palmitate (300 µM) (26). Exposure to oleate increased LD numbers, whereas exposure to palmitate did not (Fig. 1 *A* and *B*). Of the different LD regulatory proteins tested, the expression of only FIT2 was significantly reduced by palmitate (16:0) and stearate (18:0) compared with bovine serum albumin (BSA)–treated control cells. This reduction corresponded with a significant increase in C/EBP homologous protein (CHOP/DDIT3) protein levels, a major downstream proapoptotic arm of the maladaptive ER stress response. FIT2 protein reduction was not seen with chain-length, equivalent USFAs, palmitoleate (16:1), and oleate (18:1), in which levels, increased with USFA treatment (Fig. 1*C* and *SI Appendix*, Fig. S1 *A*–*D*), in line with previous observations with perilipin 2 (27). Seipin remained relatively unchanged after either SFA or USFA treatment. FIT2 protein was also reduced (threefold) in pancreatic islets from obese and diabetic *db*/*db* mice, compared with their nondiabetic littermates (in which FIT2 messenger RNA [mRNA] colocalized with insulin-positive, pancreatic β cells) (Fig. 1 *D* and *E* and *SI Appendix*, Fig. S1*E*), supporting an earlier reported reduction in β cell LD numbers in diabetic mice (16).

Next, we generated MIN6 cells lacking FIT2 (Fit2 short hairpin RNA [shRNA] cells) (*SI Appendix*, Fig. S1*F*) and mice with β cell FIT2 deletion (βFIT2KO) (*SI Appendix*, Fig. S1 *G* and *H*). FIT2 down-regulation significantly reduced LD number in both MIN6 cells (compared with scrambled, small interfering RNA [siRNA]–treated cells) (Fig. 1 *F* and *G*) and in βFIT2KO mouse islets (compared with floxed control mouse islets) (Fig. 1 *H* and *I*), the latter exacerbated by a moderate SFA western diet (saturated fat accounts for 40% of total energy). Taken together, these results suggest that the loss of FIT2 protein in β cells correlates with a failure to accumulate LDs, as observed with LDs in human β cells (16).

### Loss of β Cell FIT2 Results in Mild Glucose Intolerance.

**Fig. 2.**
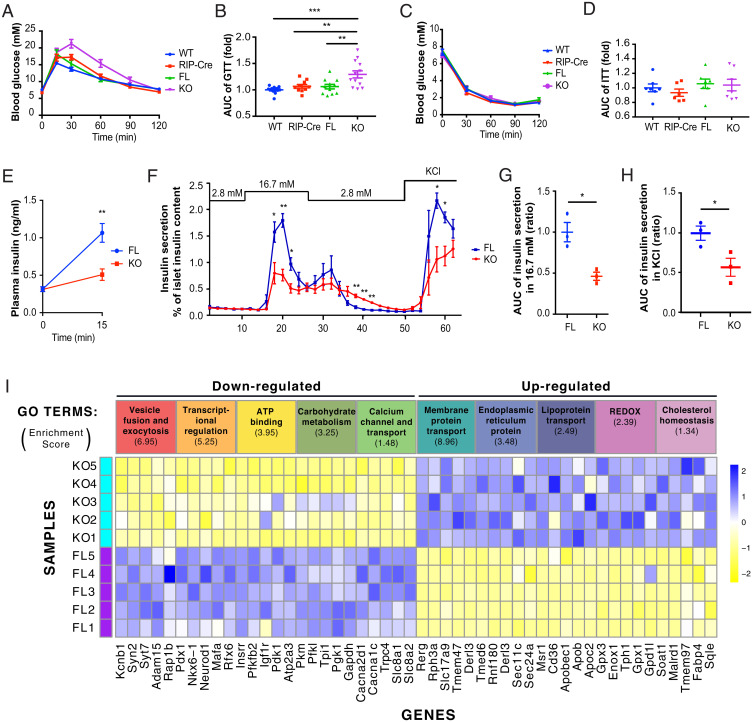
Glucose homeostasis and β cell function is impaired in βFIT2KO mice. Intraperitoneal glucose tolerance test (GTT) of age-matched, chow-fed, floxed control (FL), βFIT2KO (KO), rat insulin II promoter Cre recombinase (RIP-Cre), and wild-type (WT) mice (12- to 16-wk-old male, *n* = 10 to 13) (*A*) and corresponding area under the curve (AUC) of blood glucose levels from *A* (*B*). Insulin tolerance test (ITT) on FL, KO, RIP-Cre, and WT mice (12- to 16-wk-old male, *n* = 7) (*C*) and corresponding area under the curve (AUC) of blood glucose levels from *C* (*D*). (*E*) In vivo insulin secretion following intraperitoneal glucose administration in floxed control (FL) and βFIT2KO (KO) mice (12-wk-old male, *n* = 7). (*F*) Ex vivo islet glucose-stimulated insulin secretion from floxed control (FL) and βFIT2KO (KO) mice (12-wk-old male, *n* = 3). AUC for insulin secretion at 16.7 mM glucose (*n* = 3) (*G*) and 25 mM KCl from *F* (*H*). (*I*) GO-clustering analysis of down- and up-regulated genes in (βFIT2KO) KO as compared with floxed control (FL) islets (*n* = 5 mice). Enrichment score (≥1.3) of each GO cluster is indicated in parenthesis below each respective term, and five highly significant altered genes for each GO cluster are listed below. The full gene list is provided in Datasets S1 and S2. Values shown are mean ± SEM; *P < 0.05; **P < 0.01; and ***P < 0.001 (*B* and *D*: one-way ANOVA with Tukey’s post hoc test and *E*, *F*, *G*, and *H*: two-tailed Student's *t* test.

Given that β cell FIT2 levels were lowered following SFA exposure, we determined whether this change might impact whole-body glucose homeostasis. Here, we focused on 12-wk-old male mice. Concerns of RIP-Cre “leakiness” (28) was addressed by showing no significant change in *Fit2* mRNA expression in βFIT2KO extrapancreatic tissues (*SI Appendix*, Fig. S1*I*). There were no changes in intraperitoneal glucose tolerance test (IPGTT) and insulin tolerance test (ITT) in the control mouse lines, except for the well-documented glucose excursion increase in RIP-Cre mice (29), and yet, comparatively, 12-wk-old βFIT2KO mice displayed a mild, but significant, glucose intolerance compared with the line with the Cre transgene alone (Fig. 2 *A* and *B*).

The poorer glycemic control in βFIT2KO mice was associated with a significantly muted insulin response to high glucose, both in vivo and ex vivo (Fig. 2 *E* and *F*) and not by changes in peripheral insulin sensitivity (as measured by insulin tolerance test) (Fig. 2 *C* and *D*). There were no discernible differences in islet morphology, composition, pancreas/islet insulin content, and insulin granule morphology between βFIT2KO and floxed control mice (*SI Appendix*, Fig. S2 *A*–*F*). Rather, the muted insulin response in βFIT2KO islets could be partly attributed to an observed reduction in glucose-induced ATP/ADP ratio and a blunted, glucose-stimulated intracellular Ca^2+^ influx (*SI Appendix*, Fig. S2 *G*–*I*). Interestingly, diminished insulin secretion (Fig. 2 *F* and *H*) and Ca^2+^ influx response (*SI Appendix*, Fig. S2 *H* and *J*), following KCl-induced membrane depolarization as well as reduced exocytosis (measured by membrane capacitance), were also observed in βFIT2KO islets (*SI Appendix*, Fig. S2 *K* and *L*). This suggests that mechanisms independent of glucose metabolism may additionally contribute toward the observed reduction in insulin secretion from β cells devoid of FIT2. Comparative RNA-sequencing (RNASeq) and gene ontology (GO) analysis between islet cells from 12-wk-old chow diet–fed βFIT2KO and floxed control mice revealed distinct changes in gene clusters (Datasets S1 and S2) related to 1) vesicle fusion and exocytosis, 2) ATP binding, 3) Ca^2+^ transport, and 4) ER-related genes, as well as 5) a significant down-regulation of transcription factors linked to β cell function (Fig. 2*I* and Datasets S3 and S4). Changes in key β cell function/identity genes in islets from βFIT2KO, such as *Pdx1* and *MafA*, were corroborated using real-time qPCR (*SI Appendix*, Fig. S2*M*). Overall, these results support the view that impaired glucose tolerance in βFIT2KO mice is driven by reduced β cell function.

### Loss of FIT2 Exacerbates Diet-Induced Diabetes and Increases β Cell Ceramides and ER Stress.

**Fig. 3.**
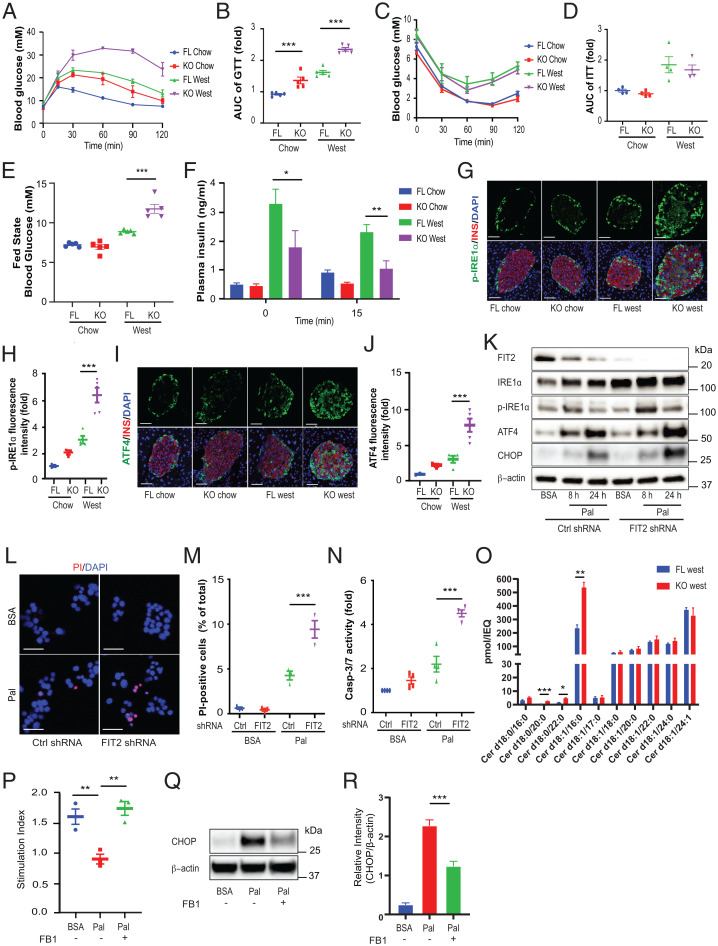
Loss of β cell FIT2 exacerbates ER stress and survival. Intraperitoneal glucose tolerance test on chow-fed, floxed control (FL, blue) and βFIT2KO (KO, red) or western diet (West)–fed, floxed control (FL, green) and βFIT2KO (KO, purple) (30-wk-old male, *n* = 5) (*A*) and area under the curve (AUC) of blood glucose levels from *A* (*B*). ITT on chow-fed, floxed control (FL, blue) and βFIT2KO (KO, red) or western diet (West) –fed, floxed control (FL, green) and βFIT2KO (KO, purple) (30-wk-old male, *n* = 4) (*C*) and AUC of blood glucose levels from *C* (*D*). (*E*) Fed state blood glucose levels of chow-fed, floxed control (FL, blue) and βFIT2KO (KO, red) or western diet (West) –fed, floxed control (FL, green) and βFIT2KO (KO, purple) (30-wk-old male, *n* = 5). (*F*) In vivo insulin secretion following glucose intraperitoneal administration in chow-fed, floxed control (FL Chow, blue) and βFIT2KO (KO Chow, red) or western diet (West) –fed, floxed control (FL West, green) and βFIT2KO (KO West, purple) (30-wk-old male, *n* = 5). (*G* and *I*) Representative immunostain for p-IRE1α and ATF4 (green), insulin (red), and DAPI (blue) in pancreas sections from floxed control (FL) and βFIT2KO (KO) mice treated with either chow or western diet (30-wk-old male). Images shown are maximum intensity projections. (Scale bar, 50 µm.) (*H* and *J*) Quantitation of relative fluorescence intensity of p-IRE1α and ATF4 staining within insulin-positive cells (*n* = 3 to 5 mice, ≥9 islets from each mouse were analyzed per group). (*K*) Representative immunoblot of indicated proteins in control shRNA-transfected or FIT2-stable knockdown MIN6 cells (FIT2 shRNA) in the absence or presence of palmitate (300 µM). (*L*) Representative image of control (Ctrl) shRNA-transfected or FIT2-stable knockdown (FIT2 shRNA) MIN6 cells in the absence (BSA only) or presence of palmitate (300 µM, 24 h) stained for DAPI (blue) and propidium iodide (PI, red). Images shown are maximum intensity projections. (Scale bar, 50 µm.) (*M*) Quantitation of the percentage of PI-positive cells over total number of cells (>1,000 cells from three independent experiments were analyzed per group). (*N*) Measurements of capase-3/7 activity in control shRNA-transfected or FIT2 knockdown (FIT2 shRNA) MIN6 cells in the absence (BSA only) or presence of palmitate (300 µM, 24 h) (*n* = 4). (*O*) Quantitation of different ceramide species in islets from western diet–fed, floxed mice (FL West) and βFIT2KO (KO West) (30-wk-old male) by LC-MS expressed as picomole per islet equivalent (pmol/IEQ) (*n* = 3). (*P*) Ex vivo insulin secretion, represented as stimulation index (16.7 mM/2.8 mM), from βFIT2KO islets treated with FB1 and palmitate. KO islets were pretreated with FB1 (10 µM) for 2 h, followed by palmitate (300 µM) in presence of FB1 for 24 h (five islets from three different mice; *n* = 3 independent experiments). (*Q* and *R*) Representative immunoblot and analysis of CHOP in MIN6 cells treated with FB1 (10 µM) in the presence and absence of palmitate (300 µM, 24 h) (*n* = 4). Values shown are mean ± SEM; *P < 0.05; **P < 0.01; and ***P < 0.001 (*P* and *R*: one-way ANOVA with Tukey’s post hoc test, *F* and *O*: two-tailed Student's *t* test [between 15 and 0 min, FL West and KO West, respectively]), and *B*, *D*, *E*, *H*, *J*, *M*, and *N*: two-way ANOVA with Šidák post hoc test.

Next, we metabolically challenged, 5-wk-old βFIT2KO mice with a high–saturated fat, high-sucrose–containing diet (western diet) for a further 25 wk. Plasma glucose concentrations following intraperitoneal bolus glucose (2 g/kg) increased in 30-wk-old, chow-fed mice, and this was significantly exacerbated in aged-matched western diet–fed βFIT2KO mice, suggesting glucose intolerance (Fig. 3 *A* and *B*). Although western diet–fed mice displayed insulin resistance, there were no significant differences between βFIT2KO mice and floxed control mice within each diet group (Fig. 3 *C* and *D*). While no significant changes in fasting-state blood glucose levels were recorded, the fed-state blood glucose levels were significantly higher in western diet–fed βFIT2KO (βFIT2 knockout [KO West]) mice compared with the floxed control mice (FIT2 floxed [FL West]) (Fig. 3*E*). Hyperinsulinemia is a well-established phenotype of diet-induced diabetes, and both western diet–fed, floxed control (FL West) and βFIT2KO (KO West) mice displayed hyperinsulinemia (Fig. 3*F*). Fasting plasma insulin levels were, however, observed to be modestly but significantly lower in βFIT2KO (KO West) mice, a difference that was more pronounced (and significant) following a glucose challenge. Hence, the loss of FIT2 in β cells did significantly compromise compensatory hyperinsulinemia, and while this was not reflected in fasting glucose levels, it was in line with the significantly elevated, fed state glucose levels of western diet–fed βFIT2KO (KO West) mice. Noteworthy, we observed no change in plasma insulin levels 15-min postglucose administration in all western diet–fed mice. Such muted, glucose-stimulated insulin responses in vivo have been described in hyperinsulinemic mice (29). The exacerbated β cell dysfunction in βFIT2KO mice exposed to a western diet was further scrutinized. We observed higher levels of p-IRE1α^+^/insulin^+^ cells and ATF4^+^/insulin^+^ cells in western diet–fed βFIT2KO (KO West) mice compared with the floxed control mice (FL West) (Fig. 3 *G*–*J*), suggesting increased, maladaptive ER stress responses in vivo. As ER stress is an important driver of β cell dysfunction (30, 31), we determined whether β cell FIT2 and LDs played a role in the palmitate-induced ER stress response. p-IRE1α was significantly elevated, with a time-dependent increase in IRE-1, ATF4, and CHOP protein levels in palmitate-treated FIT2 shRNA cells, compared with control cells (Fig. 3*K* and *SI Appendix*, Fig. S3 *A*–*D*). The amplified ER stress response (ATF4, CHOP, and IRE-1 pathways) coincided with reduced insulin secretion (*SI Appendix*, Fig. S3*E*), increased cell death (Fig. 3 *L* and *M*), and a significantly elevated activity of caspase-3/7, suggesting increased apoptosis in FIT2 shRNA cells (Fig. 3*N*). Separately, liquid chromatography–mass spectrometry (LC-MS) analysis of ceramide levels in pancreatic islets isolated from KO West and FL West (fed) mice revealed accumulation of toxic C16 ceramide (Cer d18:1/16:0) in the βFIT2KO islets (Fig. 3*O*). Similarly, there was a modest increase in the ceramide precursor C16 dihydroceramide (Cer d18:0/16:0) (*P* = 0.055) and in other dihydroceramides containing saturated fatty acyl chains, suggesting enhanced de novo ceramide synthesis in islets lacking FIT2. Accumulation of ceramides in β cells was corroborated by immunofluorescence (IF) analysis in βFIT2KO mice (*SI Appendix*, Fig. S3 *F* and *G*), and cotreatment of palmitate with fumonisin B1 (FB1), a pharmacological inhibitor of ceramide synthases, improved insulin secretion (Fig. 3*P*) in islets from βFIT2KO mice and reduced CHOP levels in palmitate-treated MIN6 cells (Fig. 3 *Q* and *R*) (32). Together, these data suggest that ceramide accumulation contributes to enhanced ER stress and dysfunction in western diet–fed βFIT2KO mice.

### Partial Restoration of FIT2 Levels Rescues LD Biogenesis and Mitigates Palmitate-Mediated Effects in MIN6 Cells.

**Fig. 4.**
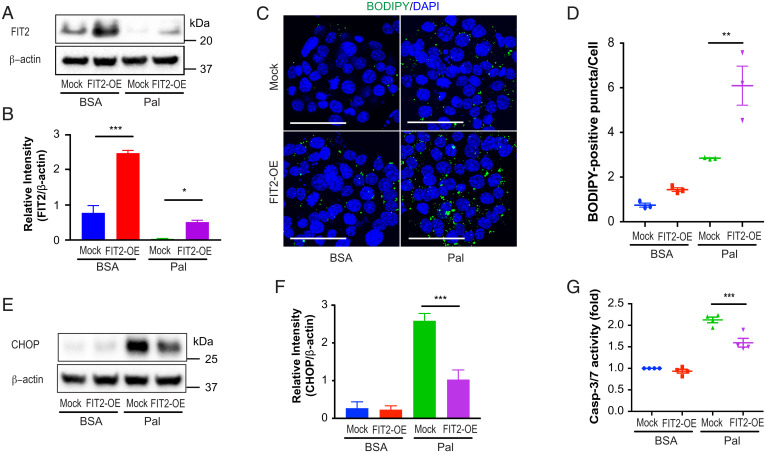
Overexpression of FIT2 in MIN6 cells ameliorates palmitate-induced lipotoxicity. (*A–G*) MIN6 cells were cultured in 6-well plates and transfected with 0.5 µg empty pcDNA3.1 vector (Mock) or pcDNA3.1-FIT2 (FIT2-OE) per well, followed by treatment with BSA or palmitate (300 µM) for 24 h. (*A* and *B*) Representative immunoblot and semiquantitation FIT2 protein levels (*n* = 3 independent experiments). (*C*) LD staining (BODIDY, green) in vector-transfected (Mock) or in FIT2-overexpressing (FIT2-OE) MIN6 cells (nuclei stained with DAPI; blue) in the absence (BSA) and presence of palmitate (300 µM, 24 h). Images shown are maximum intensity projections with a scale bar of 50 µm. (*D*) Quantitation of BODIPY-positive puncta per cell using Image J. (>1,000 cells from three independent experiments were analyzed per group). Representative immunoblot analysis (*E*) and corresponding semiquantitation of CHOP levels (*n* = 4) (*F*). (*G*) Measurements of capase-3/7 activity in control vector-transfected (Mock) or FIT2-overexpressing (FIT2-OE) MIN6 cells in the absence (BSA) or presence of palmitate (300 µM) (*n* = 4). Values shown are mean ± SEM; *P < 0.05; **P < 0.01; and ***P < 0.001 (two-way ANOVA with Šidák post hoc test).

The role of FIT2 loss in SFA-induced β cell apoptosis was further investigated through its overexpression in MIN6 cells. Cells were transiently transfected with either an expression vector–encoding FIT2 (pcDNA3.1-FIT2) or with a control vector (pcDNA3.1-mock) prior to palmitate (or BSA) exposure. FIT2 levels increased by approximately threefold in FIT2-overexpressing (FIT2-OE) cells treated with BSA compared with control (mock cells) (Fig. 4 *A* and *B*). While palmitate exposure resulted in an ∼95% reduction of FIT2 in control cells (mock transfected), this was partially attenuated in FIT-OE cells (∼50% reduction) (Fig. 4 *A* and *B*). This suggests that overexpression of FIT2 partially compensated for palmitate-induced reduction in FIT2 protein levels. This partial restoration of FIT2 in FIT-OE cells exposed to palmitate led to a significant increase in the number of LDs (Fig. 4 *C* and *D*), suggesting a partial LD rescue and that β cells LDs can be formed following palmitate exposure provided FIT2 is preserved. This rescue corresponded with a reduction in CHOP/DDIT3 protein levels and lowered caspase-3/7 activity in FIT2-OE cells exposed to palmitate (Fig. 4 *E*–*G*). Together, these results suggest that partial compensation of FIT2 loss ameliorates palmitate-induced lipotoxicity in β cells.

### Palmitoylation and ERAD-C Contribute to FIT2 Protein Loss.

**Fig. 5.**
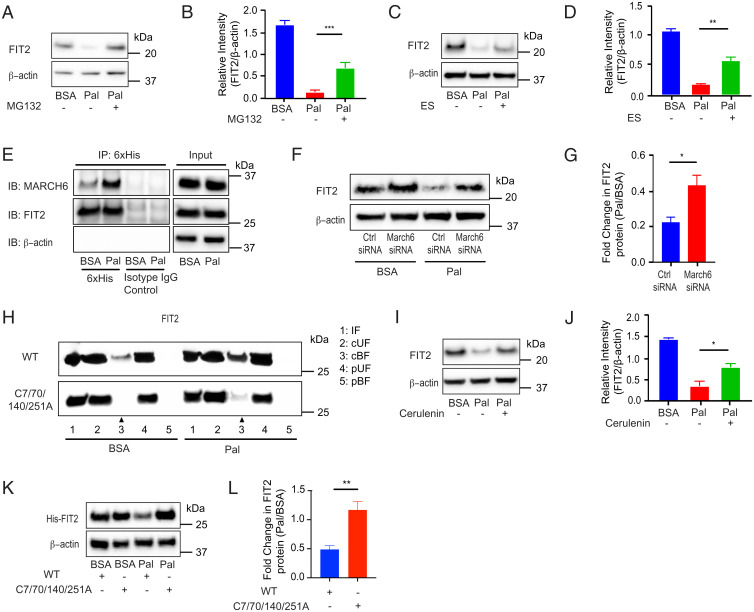
Palmitate-induced reduction of FIT2 protein involves palmitoylation with ERAD-C contributing to its degradation in MIN6 cells. (*A*–*D*) Representative immunoblot analysis and semiquantitation of FIT2 protein in MIN6 cells pretreated with respective pharmacological inhibitors 10 µM MG132 or 10 µM Eeyarestatin (ES) for 2 h, followed by cotreatment with inhibitors in the presence of palmitate (300 µM) for 4 h (*n* = 3). (*E*) Representative immunoblot (IB) for coimmunoprecipitation (IP) of FIT2 and MARCH6 in MIN6 cells in the absence (BSA) and presence of palmitate (Pal, 300 µM, 6 h), following pretreatment with 10 µM MG132 for 2 h. Pull-down of FIT2 using equal amount of input protein was carried out using 6×His antibody (with isotype control), and thereafter, captured proteins were probed for MARCH6 protein using the tGFP antibody. Representative immunoblot analysis (*F*) and semiquantitation of FIT2 protein from serum-starved MIN6 cells (2 h) transfected with scrambled control siRNA (Ctrl siRNA) or siRNA against March6 (March 6 siRNA), followed by treatment with BSA or palmitate (300 µM) for 4 h (*n* = 4) (*G*). (*H*) Representative immunoblot of S-palmitoylation assay on both wild-type (WT) and mutant (C7/70/140/251A) FIT2 under steady-state (BSA) and palmitate (Pal, 300 µM, 6 h) conditions. The increased S-palmitoylation of FIT2 under palmitate conditions (lane 3, CAPTUREome resin cleaved bound fraction [cBF]) was markedly abrogated in the mutant FIT2. Input fraction (IF) and the flowthrough fraction which represents the cleaved unbound fraction (cUF) were collected and analyzed. A separate set of protein lysates (negative control) was treated with acyl-preservation reagent and subjected to incubation with CAPTUREome resin (flowthrough fraction was used as the preserved unbound fraction [pUF], while eluted fraction was used as the preserved bound fraction [pBF]). Representative immunoblot (*I*) and corresponding semiquantitation of FIT2 protein levels in MIN6 cells pretreated with cerulenin (45 µM) for 2 h, followed by cotreatment of cerulenin (45 µM) in the presence of palmitate (300 µM) for 4 h (*n* = 3) (*J*). Representative immunoblot analysis (*K*) and corresponding semiquantitation of FIT2 protein levels in MIN6 cells transfected with pcDNA3.1-FIT2/V5-His (WT) or mutant (C7/70/140/251A), followed by treatment with BSA or palmitate (300 µM) for 24 h (*L*). Values shown are mean ± SEM; *P < 0.05; **P < 0.01; and ***P < 0.001. *B*, *D*, and *J*: one-way ANOVA with Tukey’s post hoc test and *G* and *L*: two-tailed Student's *t* test.

We next sought to elucidate the mechanism(s) that may explain SFA-induced FIT2 loss. The observed, SFA-mediated down-regulation of FIT2 in MIN6 cells was not seen at the transcriptional level (*SI Appendix*, Fig. S4*A*) but was abrogated in the presence of proteasome inhibitor MG132 (Fig. 5 *A* and *B*), suggesting that SFAs modulate FIT2 posttranslational stability. Given that FIT2 is an ER resident protein, we assessed involvement of the ER-associated degradation (ERAD) pathway. The ERAD inhibitor eeyarestatin I (33) partially, but significantly, attenuated palmitate-induced FIT2 loss (Fig. 5 *C* and *D*). Increased association between FIT2 and membrane-associated ring-CH–type finger 6 (MARCH6), a mammalian E3 ligase complex responsible for degradation of the ERAD-cytosolic substrate (ERAD-C) (34), was observed in MIN6 cells treated with palmitate (Fig. 5*E*). Furthermore, MARCH6 down-regulation (∼50%; *SI Appendix*, Fig. S4*B*), but not autocrine motility factor receptor (AMFR) down-regulation, an E3 ubiquitin ligase linked to ERAD-luminal substrate (ERAD-L) pathway (*SI Appendix*, Fig. S4 *C*–*E*), significantly rescued palmitate-mediated FIT2 loss (Fig. 5 *F* and *G*). Taken together, these results suggest ERAD-C, but not ERAD-L, contributes to palmitate-mediated FIT2 degradation.

We next tested the possibility that palmitate may directly modify FIT2, given that an in silico analysis (GPS–lipid) (35) showed high probability of fatty acid S-acylation taking place at only four cysteine sites (Cys-7, Cys-251, Cys-140, and Cys-70) (*SI Appendix*, Fig. S4*F*). S-palmitoylation on FIT2 protein was confirmed using two separate, distinct chemistry S-palmitoylation assays, with higher levels of S-acylated FIT2 proteins detected in MIN6 cells exposed to palmitate (Fig. 5*H* and *SI Appendix*, Fig. S4 *G* and *H*. Mutating all four predicted S-acylation sites (Cys to Ala) abrogated palmitate-induced modification under steady-state (BSA) and exogenous palmitate conditions (Fig. 5*H*). To confirm palmitoylation led to FIT2 degradation, cerulenin (a palmitoylation inhibitor) (36) was added together with palmitate. Cerulenin significantly reversed palmitate-mediated FIT2 protein loss (Fig. 5 *I* and *J*).

Given potential off-target effects of cerulenin (37), we tested the stability of FIT2 mutants (C7/251/140/70A) in the presence and absence of palmitate. Single mutation of either cysteine residue tended to abrogate palmitate-induced FIT2 protein loss (*SI Appendix*, Fig. S4 *I* and *J*). However, mutation of all four cysteine residues completely blocked palmitate- (Fig. 5 *K* and *L*) as well as stearate- (*SI Appendix*, Fig. S4 *K* and *L*) mediated FIT2 degradation.

## Discussion

Here, we provide evidence of a fatty acid (FA)–type, specific modulation of an ER resident, LD formation-linked protein, or FIT2 in β cells. Our results show that LD numbers are affected by the types of FA to which cells are exposed and that one pathway responsible for differential toxicity between dietary SFA and USFA (in the form of palmitate and oleate, respectively) involves cellular FIT2 protein stability and the consequent ability of β cells to accumulate LDs. We present a potential mechanism through which SFAs reduce FIT2 levels. Direct palmitoylation of FIT2 lead to increased β cell ER stress response, reduced insulin secretion, and elevated β cell apoptosis, as well as increased susceptibility to diet-induced diabetes. As FIT2 overexpression was able to rescue some LD formation and attenuate palmitate-induced ER stress, we highlight the finding that the β cell’s ability to withstand lipotoxicity is contingent on its capacity to maintain FIT2 and to generate LDs. Future studies will, however, be needed to explore the effects of FIT2 overexpression in vivo.

LDs are an important source for organelle membrane lipids and proteins (38). Mitochondrial lipid uptake from ER is essential for maintaining mitochondrial membrane integrity, which may be affected by the loss of FIT2 consistent with decreased [ATP]/[ADP] ratios (in βFIT2KO islet cells) (39). In addition to reduced cellular ATP, lowered intracellular Ca^2+^ influx was also noted in both high-glucose and high-K^+^–induced conditions, suggesting metabolic, independent changes because compromised Ca^2+^ channel activity and vesicle exocytosis (confirmed by cell capacitance recordings) were recorded in the absence of high glucose. Indeed, RNASeq analysis confirmed that, apart from reduced genes linked to carbohydrate metabolism, there were also reduction of genes linked to β cell transcription factors, vesicle fusion, and exocytosis as well as Ca^2+^ channel transport. The reduced expression of β cell transcription factors, such as *Pdx1* (pancreatic and duodenal homeobox 1) and *MafA* (V-Maf avian musculoaponeurotic fibrosarcoma oncogene homolog A), allude to a loss of β cell identity, a characteristic of ER-stressed T2D islets (40). The expression of *Kcnb1* (voltage-dependent K^+^ channel Kv2.1), *Syt7* (synaptotagmin VII), and *Rab1b* (member of RAS oncogene family) were also lower in islets of βFIT2KO mice compared with FL mice. Both KCNB1 and SYT7 play key roles in insulin exocytosis, and their depletion led to impaired insulin vesicle trafficking and secretion in β cells (41, 42). RAP1b is a small GTP-binding protein linked to accentuated insulin exocytosis by GLP-1 and glibenclamide coadministration (43). *Syn2* (syntaxin-2) expression was also lower in βFIT2KO islet cells (versus FL islet cells), but in contrast to KCNB1, SYT7, RAP1b, and all other known β cell syntaxins (e.g., SYN1A, SYN3, and SYN4), a reported loss of SYN2 increased exocytotic events in β cells (44). The action of SYN2 suggests redundancy of syntaxins in SNARE complexes. The impact of FIT2 and LD loss on β cell transcription factors and vesicle exocytosis require further investigation, especially since LDs were also shown to regulate nuclear translocation and endosomal trafficking (45).

We also observed significant elevation of ER stress–related proteins, such as p-IRE1α and ATF4 in FIT2KD cells, and in islets of βFIT2KO mice. Corroborating transcriptomic evidence of ER protein and REDOX gene up-regulation in βFIT2KO islets suggests that loss of LDs induces the maladaptive ER stress response in β cells. Accumulation of C16:0 ceramides in islets of βFIT2KO mice may also contribute to the observed ER stress and β cell dysfunction, though whether these result from changes in circulatory ceramide levels remains to be established. Nevertheless, a GENEVESTIGATOR search revealed CerS5 and CerS6 to be the most abundant ceramide synthases in pancreatic islets (46). CerS5 and CerS6 generate C16 and C14 chain-length ceramides and are likely responsible for the observed difference in C16:0 ceramide levels in western diet–fed βFIT2KO islets and floxed islets (47, 48). Further inhibition of ceramide synthesis by FB1 ameliorated palmitate-mediated ER stress in MIN6 cells and improved insulin secretion in βFIT2KO islets. When coupled with increased C16:0 ceramides levels in islets that lack FIT2, the data suggests that increased ceramides link β cell FIT2 loss with enhanced ER stress and reduced β cell function, adding to current knowledge of FIT2 loss, leading to ER lipid imbalance and ER dysregulation (23, 24).

We describe here a pathway through which palmitate drives ER stress in β cells, with FIT2 stability and LD accumulation taking center stage. Failure to sequester lipids away from the ER is likely to increase the levels of substrates for the ER-localized enzymes serine palmitoyltransferase and ceramide synthase, thus increasing ER ceramide levels (49, 50). Ceramide accumulation has been suggested to alter ER membrane properties, reduce ER protein export, disrupt ER Ca^2+^ homeostasis, and increase ER stress–mediated apoptosis (51, 52). The latter was confirmed by increased presence of CHOP, a known preapoptotic death signal, in MIN6 cells and in islets lacking FIT2. Thus, under lipotoxic conditions, FIT2 and LDs perhaps function critically to sequester and store FAs in their less toxic, esterified forms thereby preventing ceramides, especially C16:0, from accumulating within the ER.

When LDs are allowed to accumulate in β cells, for example through desnutrin (a triacylglycerol hydrolase) ablation, a similar, blunted glucose-stimulated insulin secretion (GSIS) response was observed (53). These results may seem contradictory to the current findings. However, a significant driver for dysfunction in the desnutrin knockout mice is reduced fatty acid utilization by mitochondria, rather than enhanced β cell ER stress seen in our FIT2 ablation model. So while LD formation and lipid sequestration away from the ER mitigates lipotoxicity, this is perhaps based on the assumption that LD utilization is unaffected. Here, further work is required to delineate the dynamics of LD accumulation, utilization, and turnover, especially since a higher number of LDs has been observed in human diabetic β cells (16). Nevertheless, it is increasingly clear that the formation of LDs, especially during lipotoxic conditions, is critical for preventing ER lipid accumulation and ER stress.

A fundamental mechanism governing LD loss is the posttranslational modification of FIT2 by palmitate (and stearate) and its subsequent degradation in β cells. This may explain the absence of FIT2 in genome-wide association or islet RNASeq-related studies. However, a recent type 2 diabetes meta-analysis study identified an East Asian diabetes-associated loci (RS6017317) directly in the regulatory region of FIT2 (54), and FIT2 lies adjacent to the pernicious and well-described HNF4α loci. The molecular switch of this protein, and a potential therapeutic strategy, likely involves maintaining its protein stability at the ER. Palmitoylation commonly occurs on transmembrane proteins, affecting protein stability and localization (55, 56). Interfering with palmitoylation either pharmacologically or through site-directed mutagenesis reduced palmitate-induced FIT2 degradation, and a similar mechanism was reported in other transmembrane proteins, such as TBC1D3 and CDCP1 (57, 58). We further identified ERAD-C as a contributing pathway to FIT2 degradation. We found increased MARCH6 and FIT2 protein association in the presence of palmitate and reduced FIT2 degradation in MARCH6 knockdown cells, but not in AMFR (ERAD-L) knockdown cells. Mutating predicted, S-acylated cysteine residues abrogated FIT2 palmitoylation and degradation. These results suggest that SFA-mediated S-acylation (palmitoylation) of the FIT2 protein promotes its degradation through a mechanism which involves, but is perhaps not limited to, the ERAD-C pathway. Although we show that the ERAD-L pathway is unlikely to be involved, we do not exclude the involvement of other protein degradation mechanisms. This link between SFA-mediated β cell lipotoxicity and FIT2 degradation impacting LD numbers has not been described previously. Further molecular work correlating the degree of FIT2 palmitoylation with its degradation in both physiology and pathology is required to further our understanding on how FIT2 function is fine-tuned at the protein level.

In conclusion, our results describe a means through which SFAs may disable LD biogenesis in β cells. Palmitoylation of FIT2 leads to its degradation in part through ERAD-C–mediated mechanisms. Beyond FIT2 and the understanding of how SFAs are more damaging than USFAs to cells, our results show that restoration of LD formation, especially in a lipotoxic milieu, such as obesity and diabetes, is of considerable therapeutic value for preventing β cell dysfunction and loss.

## Materials and Methods

### Mice.

β cell–specific FIT2 knockout mice (βFIT2KO, KO) were generated using the Cre-lox recombination system. Mice with floxed FIT2 (FIT2_fl/fl_, FL) (21) were bred with mice expressing Cre-recombinase, under control of the rat insulin promoter [B6.Cg-Tg(Ins2-cre)25Mgn/J]. Male mice were used in all experiments. βFIT2KO and their corresponding, floxed littermates (FIT2_fl/fl_) were fed with western-type (West) diet (D12079B, Research Diets) from 5 wk of age for a further 25 wk. B6.BKS (D)-Leprdb/J (db/db) mice were compared with their age-matched, heterozygous littermates (db/+) at 16 wk. Mice were housed in a facility with a 12-h light–dark cycle and with food and water available ad libitum. All the animal experiments and protocols were approved by the Institutional Animal Care and Use Committee of SingHealth (IACUC SingHealth No. 2013/SHS/816) and of Nanyang Technological University Singapore (A0373).

### Islet Isolation, Dispersion, and Cell Culture.

Mouse pancreatic islets were isolated by perfusing the pancreas through the common bile duct with collagenase, as previously described (59). The isolated islets were dispersed into single cells by incubation with Accutase at 37 °C for 3 to 5 min. MIN6 cells (passages 30 to 35) were cultured as previously described (60). Transient and stable transfections of MIN6 were performed (*SI Appendix*). FAs (palmitate, stearate, oleate, and palmitoleate) were conjugated with FA-free BSA at a 2:1 molar ratio and used at a final concentration of 300 μM.

### Immunoblotting and IF Assays.

Cells and isolated islets were lysed in radioimmunoprecipitation assay (RIPA) buffer supplemented with protease inhibitor mixture. Proteins were separated by sodium dodecyl sulfate–polyacrylamide gel electrophoresis and transferred onto nitrocellulose membranes with blocking, antibody incubation, and image acquisition (*SI Appendix*). For IF, cryosections of 10 µm thickness from fixed and cryopreserved pancreas were used for IF analysis. Sections were rinsed with Tris-buffered saline (TBS), permeabilized, blocked with 10% normal goat serum plus 0.2% Triton X-100 in TBS for 1 h at room temperature, and incubated overnight with primary antibodies at 4 °C in a humidified atmosphere (*SI Appendix*).

### RNA Extraction, qRT-PCR, RNASeq Library Preparation, and Data Processing.

Total RNA was extracted and reverse transcribed according to the manufacturer’s instructions (*SI Appendix*). Real-time PCR was performed on QuantStudio 6 Flex Real-Time PCR System (Applied Biosystems) using Power SYBR Green PCR Master Mix (Applied Biosystems), with intron-spanning primers (Table S1). Pancreatic islets were isolated from mice in quintuplicate. Following overnight recovery, total RNA was harvested using followed by RNASeq library construction (*SI Appendix*). Genes were considered to be significantly and differentially expressed when false discovery rate ≤ 0.05, with fragments per kilobase of exon per million mapped fragments ≥ 1 in one sample group retained for subsequent analysis. GO-clustering enrichment analysis was carried out on differential genes using the Functional Annotation tool in DAVID version 6.8 (61, 62), under medium stringency for all default annotation categories except protein domains. We identified significant, clustered groups having group enrichment scores of ≥1.3, with higher scores indicative of more significant, annotated GO clusters.

### Insulin, Intracellular Ca^2+^, and Membrane Capacitance Measurements.

MIN6 cells and pancreatic islets were preincubated at 37 °C for 2 h in low-glucose Krebs ringer bicarbonate buffer prior to insulin secretion assay (*SI Appendix*). To determine islet insulin content, 10 isolated islets were washed twice with ice-cold phosphate-buffered saline and then lysed with RIPA buffer. Insulin and protein content of the lysate were measured using mouse insulin enzyme-linked immunosorbent assay (ELISA) (Mercodia) and bicinchoninic acid assay (Thermo Fisher Scientific), respectively. Pancreatic insulin content was determined following acid extraction (*SI Appendix*). Changes in intracellular Ca^2+^ and membrane capacitance were recorded from intact pancreatic islets using fura2-AM on a fluorescence, wide-field microscope and a standard, whole-cell patch clamp technique, respectively (*SI Appendix*). For in vivo glucose–stimulated insulin secretion and ITT, mice were starved for 6 h with ad libitum access to water and then administered glucose (2 g/kg) or insulin (0.75 unit/kg), respectively. Blood samples were collected from the tail vein prior to, and 15 min after, intraperitoneal injection. Blood glucose was measured in blood samples collected from tail vein using a glucometer (Accu-Chek Performa Nano System). Total blood was centrifuged at 1,000 × *g* for 10 min, and the supernatant (serum) was collected for insulin measurements using mouse insulin ELISA (Mercodia).

### Ceramides Analysis.

Pancreatic islets were prepared (*SI Appendix*) and analyzed using an Agilent 1290 series Ultra-high-performance liquid chromatography system connected to an Agilent 6495 QQQ mass spectrometer after separation on a ZORBAX Eclipse plus C18 column (2.1 × 50 mm, 1.8 µm, 95 Å, Agilent) at 40 °C (*SI Appendix*). Mass spectrometry analysis was performed in positive ion mode with dynamic, scheduled multiple reaction monitoring (MRM). Mass spectrometry settings, LC-MS gradient, and MRM transitions for each lipid class were adapted from a previously published method (63). Data analysis was performed on Agilent MassHunter Quantitative analysis software. Relative quantitation was based on one-point calibration with Ceramide d18:1/8:0. The data were further normalized to the total number of islets in each sample.

### Coimmunoprecipitation, S-acylation, and S-palmitoylation Assays.

MIN6 cells, transiently transfected with pcDNA3.1-FIT2/V5-His, were pretreated with 10 µM MG132 (Cell Signaling Technology) for 2 h and thereafter treated with 300 µM palmitate (BSA-conjugated) for 6 h. For coimmunoprecipitation, GFP-tagged MARCH6 (BC059190) mouse-tagged open reading frame Clone (Origene) was cotransfected with pcDNA3.1-FIT2/V5-His prior to pretreatment (*SI Appendix*). S-palmitoylation assay was performed using CAPTUREome S-Palmitoylated Protein Kit (Badrilla), in accordance with manufacturer’s instruction (*SI Appendix*). For Click-iT Palmitoylation assay, MIN6 cells, transiently transfected with a 6HIS-tagged FIT2 overexpression plasmid, were pretreated with 10 µM MG132 (Cell Signaling Technology) for 2 h; then treated with 200 µM Click-IT Palmitic Acid, Azide (Thermo Fisher Scientific) for 6 h; and thereafter assay was carried out according to manufacturer’s recommendation without modifications (*SI Appendix*).

### Statistical Analysis.

Data tested for normality (Shapiro–Wilk test) are expressed as mean ± SEM. Appropriate statistical analysis using either Student’s *t* test, one-way ANOVA with Tukey’s post hoc test, or two-way ANOVA with Šidák post hoc test were used to determine statistical difference, and *P* values < 0.05 were considered as statistically significant (Prism 7, GraphPad).

## Supplementary Material

Supplementary File

Supplementary File

Supplementary File

Supplementary File

Supplementary File

## Data Availability

RNASeq data reported in this paper are publicly available at NCBI Gene Expression Omnibus (accession no. GSE133939) (64). Authors declare no primary datasets and computer codes linked to this study. All other study data are included in the article and/or supporting information.
