# Supplementary material for: Destabilization of β Cell FIT2 by saturated fatty acids alter lipid droplet numbers and contribute to ER stress and diabetes
Source: Proc Natl Acad Sci U S A. 2022 Mar 7;119(11):e2113074119. doi: 10.1073/pnas.2113074119 (PMC8931238; doi:10.1073/pnas.2113074119)
Supplement: Supplementary File [file pnas.2113074119.sapp.pdf]

Fig S1

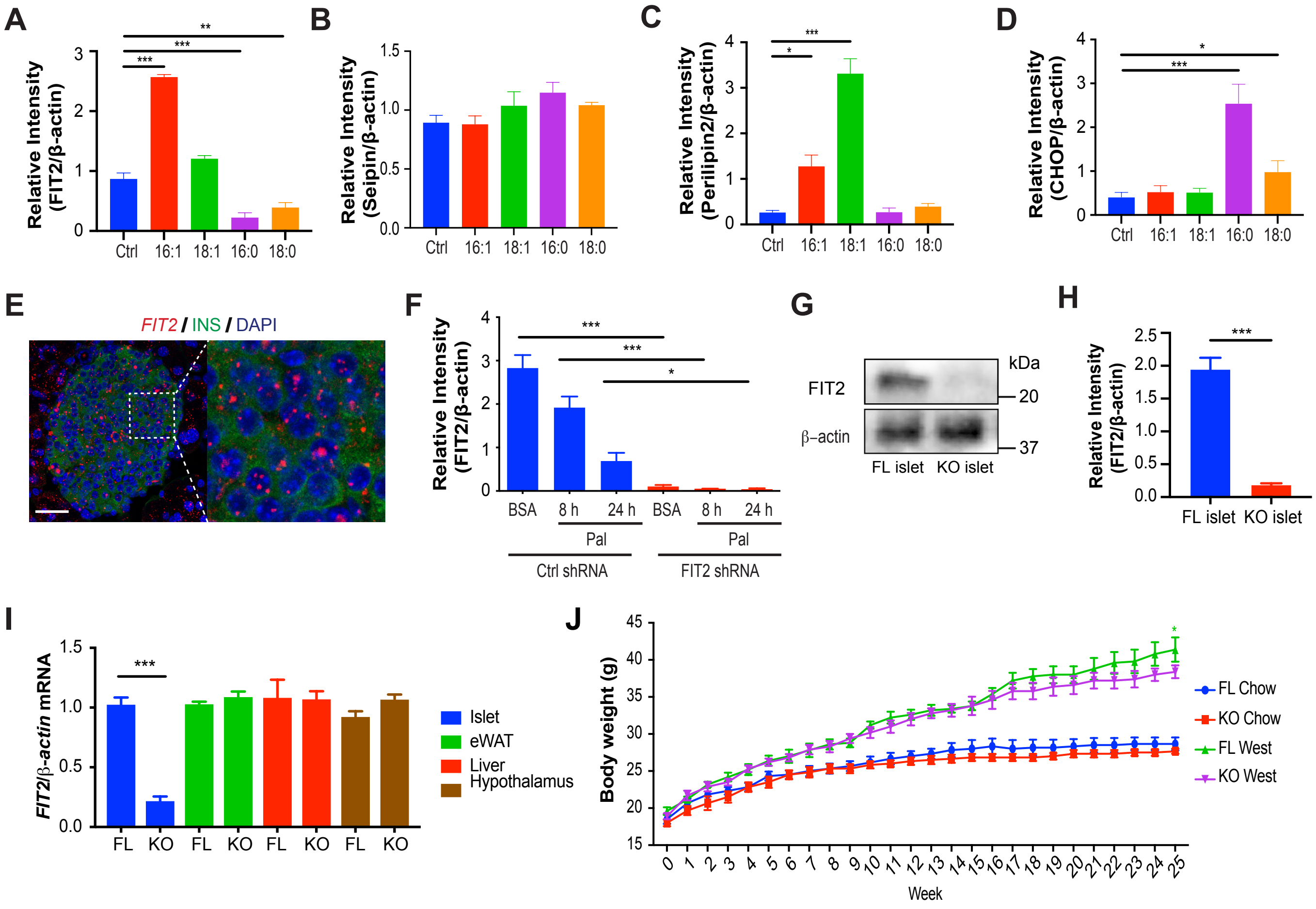

A
Relative Intensity (FIT2/β-actin)
Ctrl 16:1 18:1 16:0 18:0
B
Relative Intensity (Seipin/β-actin)
C
Relative Intensity (Perilipin2/β-actin)
D
Relative Intensity (CHOP/β-actin)
E
FIT2 / INS / DAPI
F
Relative Intensity (FIT2/β-actin)
BSA 8 h 24 h BSA 8 h 24 h
Pal
Ctrl shRNA
FIT2 shRNA
G
FIT2
β-actin
kDa
20
37
FL islet KO islet
H
Relative Intensity (FIT2/β-actin)
FL islet KO islet
I
FIT2/β-actin mRNA
FL KO FL KO FL KO FL KO
Islet
eWAT
Liver
Hypothalamus
J
Body weight (g)
Week
FL Chow
KO Chow
FL West
KO West

Fig S2

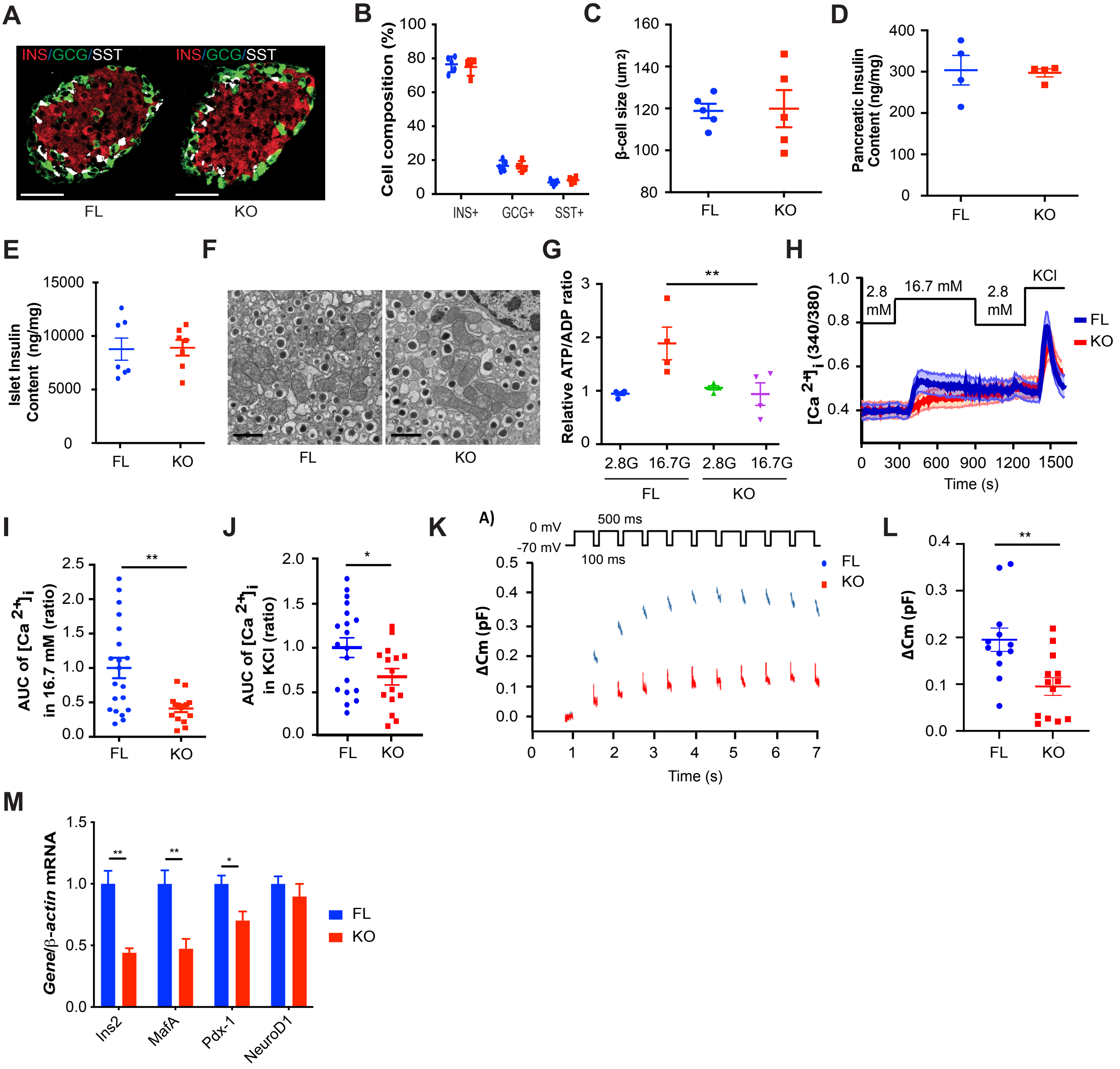
A
INS/GCG/SST
INS/GCG/SST
FL
KO
B
Cell composition (%)
100
80
60
40
20
0
INS+
GCG+
SST+
C
β-cell size (um2)
160
140
120
100
80
FL
KO
D
Pancreatic Insulin Content (ng/mg)
400
300
200
100
0
FL
KO
E
Islet Insulin Content (ng/mg)
15000
10000
5000
0
FL
KO
F
FL
KO
G
Relative ATP/ADP ratio
**
3
2
1
0
2.8G
16.7G
2.8G
16.7G
FL
KO
H
[Ca 2+]i (340/380)
1.0
0.8
0.6
0.4
2.8 mM
16.7 mM
2.8 mM
KCl
FL
KO
0
300
600
900
1200
1500
Time (s)
I
AUC of [Ca 2+]i in 16.7 mM (ratio)
**
2.5
2.0
1.5
1.0
0.5
0.0
FL
KO
J
AUC of [Ca 2+]i in KCl (ratio)
*
2.0
1.5
1.0
0.5
0.0
FL
KO
K
A)
0 mV
500 ms
-70 mV
100 ms
FL
KO
ΔCm (pF)
0.5
0.4
0.3
0.2
0.1
0.0
0
1
2
3
4
5
6
7
Time (s)
L
ΔCm (pF)
**
0.4
0.3
0.2
0.1
0.0
FL
KO
M
Gene/β-actin mRNA
1.5
1.0
0.5
0.0
**
**
*
Ins2
MafA
Pdx-1
NeuroD1
FL
KO

Fig S3

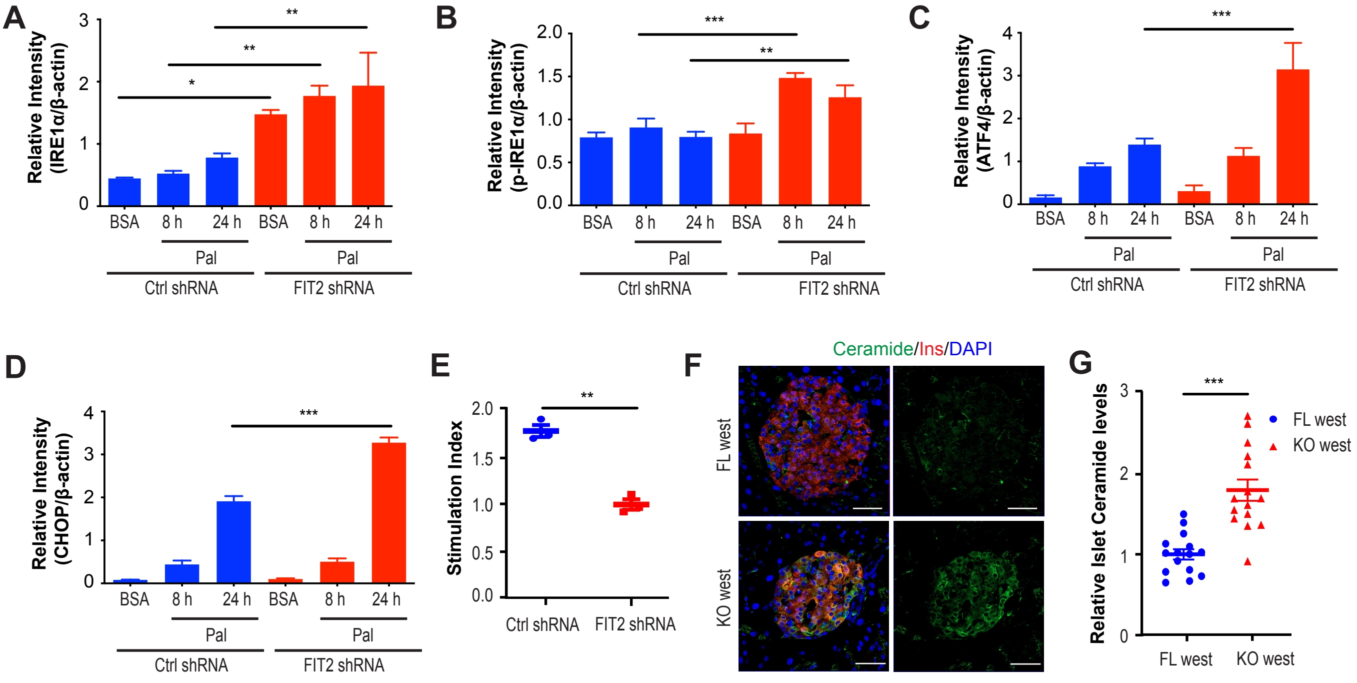
A
Relative Intensity (IRE1α/β-actin)
3
2
1
0
*
**
**
BSA
8 h
24 h
BSA
8 h
24 h
Pal
Pal
Ctrl shRNA
FIT2 shRNA
B
Relative Intensity (p-IRE1α/β-actin)
2.0
1.5
1.0
0.5
0.0
***
**
BSA
8 h
24 h
BSA
8 h
24 h
Pal
Pal
Ctrl shRNA
FIT2 shRNA
C
Relative Intensity (ATF4/β-actin)
4
3
2
1
0
***
BSA
8 h
24 h
BSA
8 h
24 h
Pal
Pal
Ctrl shRNA
FIT2 shRNA
D
Relative Intensity (CHOP/β-actin)
4
3
2
1
0
***
BSA
8 h
24 h
BSA
8 h
24 h
Pal
Pal
Ctrl shRNA
FIT2 shRNA
E
Stimulation Index
2.0
1.5
1.0
0.5
0.0
**
Ctrl shRNA
FIT2 shRNA
F
Ceramide/Ins/DAPI
FL west
KO west
G
Relative Islet Ceramide levels
3
2
1
0
***
FL west
KO west
FL west
KO west

Fig S4

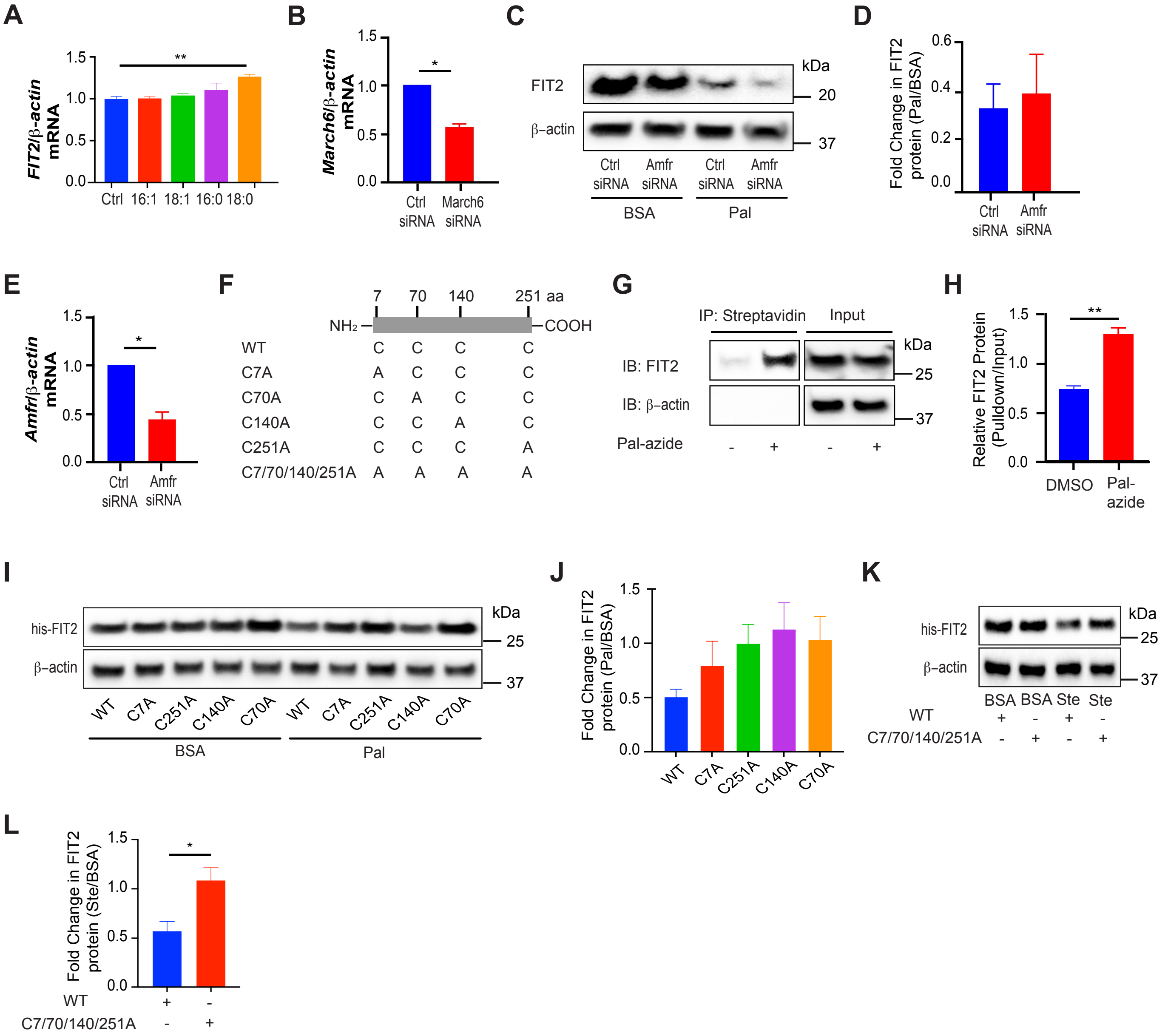

A
FIT2/β-actin mRNA
Ctrl 16:1 18:1 16:0 18:0
**
B
March6/β-actin mRNA
Ctrl siRNA March6 siRNA
*
C
FIT2
β-actin
kDa
20
37
Ctrl siRNA Amfr siRNA Ctrl siRNA Amfr siRNA
BSA
Pal
D
Fold Change in FIT2 protein (Pal/BSA)
Ctrl siRNA Amfr siRNA
E
Amfr/β-actin mRNA
Ctrl siRNA Amfr siRNA
*
F
7 70 140 251 aa
NH2 COOH
WT C C C C
C7A A C C C
C70A C A C C
C140A C C A C
C251A C C C A
C7/70/140/251A A A A A
G
IP: Streptavidin Input
IB: FIT2
IB: β-actin
kDa
25
37
Pal-azide - + - +
H
Relative FIT2 Protein (Pulldown/Input)
DMSO Pal-azide
**
I
his-FIT2
β-actin
kDa
25
37
WT C7A C251A C140A C70A WT C7A C251A C140A C70A
BSA
Pal
J
Fold Change in FIT2 protein (Pal/BSA)
WT C7A C251A C140A C70A
K
his-FIT2
β-actin
kDa
25
37
BSA BSA Ste Ste
WT + - + -
C7/70/140/251A - + - +
L
Fold Change in FIT2 protein (Ste/BSA)
*
WT + -
C7/70/140/251A - +

Supplementary Information (SI) Appendix

SUPPLEMENTARY FIGURE LEGENDS

Supplementary Fig 1: Relative levels of LD formation proteins following FA treatment as well as analysis of FIT2 in islets of βFIT2KO mice.
A-D, Semi-quantitation of immunoblots for FIT2, Seipin, Perilipin2 and CHOP protein levels in MIN6 cells treated with palmitoleate (16:1), oleate (18:1), palmitate (16:0), stearate (18:0) (300 μM, 24 h) or BSA control (N=3). **E**, Representative *in situ* hybridization image for *FIT2* mRNA (red) combined with immunohistochemistry for insulin (green) in pancreatic cryosections from C57BL/6 mice. DAPI was used to counterstain the cell nucleus (blue). Expanded image (right) of the demarcated section. Images shown are maximum-intensity projections. Scale bar = 50 μm. **F,** Semi-quantitation of immunoblot analysis of FIT2 expression in vector only (Ctrl shRNA) or stable FIT2 knockdown (FIT2 shRNA) MIN6 cells treated with or without palmitate (300 μM, 24 h). **G**, Representative immunoblot and **H**, corresponding semi-quantitation of FIT2 protein in isolated islets from floxed control (FL) and βFIT2KO (KO) mice (12 wk-old male, N=7). **I**, Gene expression (qPCR) analysis of *FIT2* mRNA levels in pancreatic islets, adipose tissue, liver and hypothalamus of floxed control (FL) and βFIT2KO (KO) mice (12 wk-old male, N=3-4). **J**, Body weight of floxed control (FL) and βFIT2KO (KO) fed with chow diet (FL Chow, KO Chow) or West diet (FL West, KO West) for 25 weeks (30 wk-old at time of analysis, male, N=6). Values shown are mean ± SEM; *, $P < 0.05$; **, $P < 0.01$; ***, $P < 0.001$ relative to control (A, B, C, D: one-way ANOVA with Tukey's post-hoc test, F, J: two-way ANOVA with Šidák post-hoc test), H, I: two-tailed Student's T-test.

Supplementary Fig 2: Islet characteristics of βFIT2KO mice.
A, Representative immunostaining for insulin (red), glucagon (green) and somatostatin (white) in pancreas sections from FL and KO mice (12 wk-old male). Scale bar = 50 μm. **B, C,** Cell-type distribution and β-cell size analysis of *in situ* islets in pancreas sections from floxed control (FL) and βFIT2KO (KO) mice (12 wk-old male, N=4-5). **D, E**, Measurements of total pancreas (N=4) and islet (N=7) insulin content. **F**, Representative Transmission Electron Microscope (TEM) images of insulin granules of FL and KO pancreata (N=3). Scale bar = 1 μm. **G**, ATP/ADP ratio of FL and KO islets in 2.8 mM glucose (2.8 G) followed by stimulation with 16.7 mM glucose (16.7 G). (N=4). **H**, Measurements of changes in $[Ca^{2+}]_i$ in FL and KO islets exposed to varying concentrations of glucose "G" as indicated (15-20 islets from four independent experiments were analyzed per group). **I**, AUC of $[Ca^{2+}]_i$ at 16.7 mM glucose (N=15-20). **J**, AUC of $[Ca^{2+}]_i$ at 25 mM KCl (N=15-20). L, K, Measurements of capacitance change in FL and KO islets. L, Representative traces of capacitance recording obtained with a train of 10 consecutive depolarising pulses (500 ms steps, 100 ms interval) from -70 mV to 0 mV on FL (blue) and KO (red) islets. K, Quantification of changes in capacitance upon the 10th depolarising pulse induction between FL and KO islets (N=12 islets from 3 different 12-wk old mice each). **M**, qPCR validation of RNA-Seq results with respect to changes seen in key β-cell genes (N=5). Values shown are mean ± SEM; *, $P < 0.05$; **, $P < 0.01$; ***, $P < 0.001$ (B, C, D, E, I, J, L, M: two-tailed Student's T-test, G: two-way ANOVA with Šidák post-hoc test).

Supplementary Fig 3: Relative levels of ER stress proteins and ceramide immunodetection in pancreas of βFIT2KO mice.

A-D, Semi-quantitation of immunoblot analysis of IRE1α, p-IRE1α, ATF4 and CHOP protein levels in scrambled (Ctrl shRNA) or FIT2 knockdown (FIT2 shRNA) MIN6 cells in the absence (BSA) or presence of palmitate (300 μM, 24 h). **E**, *Ex vivo* insulin secretion, represented as Stimulation Index (16.7 mM / 2.8 mM), from scrambled (Ctrl shRNA) or FIT2 knockdown (FIT2 shRNA) MIN6 cells. **F,** Representative immunostaining image for insulin (red), ceramide (green) and DAPI (blue) in pancreas sections from western diet fed FL and KO mice (30 wk-old, male, N=3). Scale bar = 50 μm. **G,** Quantitation of ceramide fluorescence intensities from floxed control (FL) and βFIT2KO (KO) islets of western diet fed mice (30 wk-old, male, N=3) (normalised to FL) using Image-J. Values shown are mean ± SEM; *, P < 0.05; **, P < 0.01; ***, P < 0.001 (A, B, C, D: two-way ANOVA with Šidák post-hoc test, E, G: two-tailed Student's T-test.

Supplementary Fig 4: Relative changes in gene and protein levels of FIT2 following exposure to different FAs and separately on MARCH6 and AMFR knockdown in MIN6 cells.

A, Relative *FIT2* mRNA levels (qPCR) in MIN6 cells treated with BSA or different FAs (300 μM) for 24 h (N=3). **B-E**, MIN6 cells were transiently transfected with a scrambled (ctrl siRNA) or different siRNA oligonucleotides targeting either *March6* or *Amfr.* 48 h after transfection, cells were serum-starved for 2 h followed by treatment with either BSA or palmitate (300 μM) for 4 h. **B,** Relative *March6* gene expression levels following siRNA mediated knockdown. **C, D,** Changes in FIT2 protein following AMFR knockdown and palmitate (300 μM, 4h) exposure. **E,** Relative *Amfr* gene expression levels following siRNA mediated knockdown. **F,** Schematic representation of point mutants (C ➔ A) of FIT2. **G, H**, Click-iT palmitoylation assay of FIT2 protein in MIN6 cells transiently transfected with pcDNA3.1-FIT2/V5-His and pre-treated with 10 μM MG132 for 2 h followed by DMSO (Vehicle control) or 200 μM Click-IT™ Palmitic Acid, Azide treatment for 6 h. **I, J**, Representative immunoblot analysis and semi-quantification of FIT2 protein levels in MIN6 cells transfected with pcDNA3.1-FIT2/V5-His (WT) or mutants followed by treatment with BSA or palmitate (300 μM, 24 h). **K,** treatment of wild-type or quadruple mutant FIT2 with stearate (300 μM) for 24 h (N=3-4). Values shown are mean ± SEM; *, P < 0.05; **, P < 0.01; ***, P < 0.001 (A, J: one-way ANOVA with Tukey's post-hoc test, B, D, E, H, L: two-tailed Student's T-test.

SUPPLEMENTARY MATERIALS AND METHODS

Islet isolation, dispersion and cell culture

Mouse pancreatic islets were isolated by perfusing the pancreas through the common bile duct with collagenase. The isolated islets were dispersed into single cells by incubation with Accutase at 37°C for 3-5 min. The primary islet cells were cultured in CMRL medium supplemented with 10% heat-inactivated fetal bovine serum (FBS), 100 U/ml penicillin and 100 µg/ml streptomycin. The mouse pancreatic β-cell line MIN6 was kindly provided by Dr. Jun-ichi Miyazaki, Osaka University, Japan (1). MIN6 cells (passages 30–35) were cultured in DMEM containing 25 mM glucose,10% heat-inactivated fetal bovine serum, 4 mM L-glutamine, 100 U/ml penicillin, 100 µg/ml streptomycin and 50 µM 2-mercaptoethanol. HEK 293T/17 cells were cultured in Dulbecco's modified Eagles medium (DMEM, 25 mM glucose) containing 10% fetal bovine serum, 4 mM L-glutamine, 100 U/ml penicillin and 100 µg/ml streptomycin. Cells were maintained at 37 °C under 5% CO2 and 95% air condition. Transient transfections of Min6 and HEK 293T/17 cells were performed using Liofectamine 2000 (Thermo Fisher Scientific) according to the manufacturer's instructions. FFAs (palmitate, stearate, oleate and linoleate) were conjugated with FFA-free BSA at a 2:1 molar ratio and used at a final concentration of 300 µM.

Lentiviral transduction for generation of shRNA-mediated stable knockdown cell lines.

MISSION lentiviral transduction particles expressing shRNA targeting FIT2 (SHCLNV-NM_173397) and lentiviral-negative control particles (SHC002V) were purchased from Sigma-Aldrich. Stable transduced cell lines were generated according to the manufacturer's instructions. Briefly, cells were seeded on a 12-well plate and infected with the lentiviral particles the following day. After 24 h medium was changed. Selection of lentiviral expressing cells started 24 h later with the addition of 2 µg/ml puromycin. Knockdown efficiency of different cell lines was determined by immunoblotting and real-time PCR.

Protein extraction and immunoblotting assays

Cells and isolated islets were lysed in RIPA buffer supplemented with protease inhibitor cocktail. Proteins were separated by SDS-PAGE and transferred onto nitrocellulose membranes. Blocking was performed at room temperature for 1 h in Tris-buffered saline (TBS) with 5% non-fat milk, followed by incubation with the different primary antibodies (described below) in TBS with 5% non-fat milk for either 1 h at room temperature or overnight at 4 °C. After several washes with TBS containing 0.5% Tween 20 (TBST), the membranes were incubated with secondary antibodies of anti-mouse/rabbit IgG/HRP (as appropriate) in TBS with 1% non-fat milk. Following several washes, the protein bands were visualized using enhanced chemiluminescence (Cell Signaling Technology) and quantified using ImageJ.

RNA extraction and quantitative RT-PCR

Total RNA was prepared from tissues or cells using NucleoSpin RNA II kit (Macherey-Nagel), or prepared from the isolated islets using the RNeasy® Plus Mini Kit (Qiagen). 1 µg of RNA was reversely transcribed using High Capacity cDNA Reverse Transcription Kit (Applied Biosystems) according to the manufacturer's instructions. Real-time PCR was performed on QuantStudio 6 Flex Real-Time PCR System (Applied Biosystems) using Power SYBR Green PCR Master Mix (Applied

Biosystems). The PCR primers used are summarized in Supplementary Table 3.

RNA-seq library preparation

Pancreatic islets were isolated from 12 week old wild type (WT) and β-cell specific FIT2 knockout (β-FIT2KO, KO) mice in quintuplicate. Isolated pancreatic islets then cultured in complete CMRL medium overnight for recovery. Total RNA was harvested using RNeasy Plus Mini Kit (Qiagen). RNA-seq library construction was conducted by Seqmatic. Briefly, polyA-tail specific mRNA libraries were prepared from 1 ug of total RNA with Illumina TruSeq stranded mRNA library preparation protocol followed by single-end 75bp sequencing with the Illumina NextSeq to generate an average of 50,578,630 reads per sample.

RNA-Seq data processing

Sequenced reads were aligned to the mm10 (GRCm38) mouse genome with STAR version 2.5.2a (2) and transcripts were assembled with RSEM version 1.3.0 (3). Cuffnorm version 2.2.1 (4, 5) was used to obtain quartile-normalized fragments per kilobase per million reads (FPKM) expression matrix of genes across all samples. Cuffdiff2 version 2.2.1 (4, 5) was used to identify differential genes between samples. Genes were considered to be significantly differentially expressed when false discovery rate (FDR) $\leqslant$ 0.05, with FPKM $\geqslant$ 1 in one sample group retained for subsequent analysis. GO clustering enrichment analysis was carried out on differential genes using the Functional Annotation tool in DAVID version 6.8 under medium stringency for all default annotation categories except protein domains. We identified significant clustered groups having group enrichment scores of $\geqslant$ 1.3, with higher scores indicative of more significant annotated GO clusters. Heatmaps were generated with R package pheatmap version 1.0.10 (https://cran.r-project.org/web/packages/pheatmap/index.html).

Immunofluorescence

Pancreata were fixed with 4% paraformaldehyde for 6 h, followed by incubation with 30% sucrose overnight at 4 °C. Cryosections of 10 μm thickness were used for immunofluorescence analysis. Sections were rinsed with TBS, permeabilized, and blocked with 10% normal goat serum plus 0.2% Triton X-100 in TBS for 1 h at room temperature and then incubated overnight with primary antibodies at 4°C in a humidified atmosphere. After gentle washing with TBS and incubating with fluorescence secondary antibodies for 1 h at room temperature, sections were mounted with Vecta Mount solution (Vector Labs) and analysed by confocal imaging (Leica). For LD staining, cryosections were stained with BODIPY 493/503 (0.01mg/ml) and DAPI (5 μg/ml) for 15 min at RT. For propidium iodide (PI) staining, cells were incubated with 10 μg/ml PI and 5 μg/ml DAPI in medium for 1h at 37 °C followed by RT fixation. Images were captured using a SP8 confocal microscope (Leica). BODIPY-positive puncta per cell and percentage of PI-positive cells were quantified using Image-J and Prism 7 (GraphPad).

LD staining

Cells were fixed with 4% paraformaldehyde solution for 15 min at RT. Fixed cells or pancreas cryosections of 10 μm thickness were stained with BODIPY 493/503 (0.01mg/ml) and DAPI (5 μg/ml) for 15 min at RT. Images were captured using a SP8 confocal microscope (Leica). BODIPY-positive puncta per cell was qauntified using Image-J and Prism 7 (GraphPad).

Propidium Iodide staining

Cells were incubated with 10 µg/ml propidium iodide (PI) and 5 µg/ml DAPI in medium for 1h at 37 °C. After 3 times washing with phosphate-buffered saline (PBS), cells were fixed with 4% paraformaldehyde solution for 15 min at RT and subjected to confocal imaging. Percentage of PI-positive cells was quantified using Image-J Prism 7 (GraphPad).

Islet cell composition and beta β-cell size

Islet cell composition was determined by dividing the number of individual cell types (α, β or δ cells) by the total islet cell number per islet. β-cell size was determined by dividing the insulin-positive area of each islet by the number of insulin-positive cells within the same islet. For each group, >40 islets from non-serial sections of pancreatic tissues from 5 animals were analyzed for both β-cell size and islet cell composition.

Transmission electron microscopy

Mouse pancreas were either directly fixed at room temperature using 4% formaldehyde/2% glutaraldehyde (Electron Microscopy Sciences, Hatfield, PA) in 0.1 M sodium cacodylate, pH 7.4, or cryo-fixed overnight as described previously (47). Tissue samples were then washed twice with 0.1 M cacodylate buffer, post-fixed for 2 h in a mixture consisting of 1% osmium tetroxide (EMS) and 1.5% potassium ferrocyanide (EMS) in 0.1M cacodylate, pH 7.4. Post-fixed tissues were washed with distilled H2O and dehydrated through an ethanol series (25%, 50%, 75%, 85%, 90%, 95%, 100%) and then in propylene oxide (EMS). Samples were subsequently embedded in Epoxy resin for 1 h at 42℃ followed by 48 h at 65℃. For all samples, ultrathin sections of 50 nm were cut with diamond knife (Diatome, Switzerland) on Leica Ultracut EM UC7 ultramicrotome (Leica Microsystems, Germany) and collected onto formvar/carbon copper slot grids (EMS). Sections on grids were contrasted with 4% aqueous uranyl acetate followed by Reynold's lead citrate and analysed with a JEM-1010 Jeol transmission electron microscope (JEOL Ltd., Japan) operating at 80 kV. Images were acquired with SIA model 12C high resolution full-frame CCD camera.

Total islet and pancreatic insulin content

To determine islet insulin content, around 10 isolated islets were washed twice with ice-cold D-PBS and then lysed with RIPA buffer. Insulin and protein content of the lysate were measured using Mercodia Mouse Insulin ELISA (Mercodia) and BCA assay (Thermo Fisher Scientific), respectively. Total islet insulin content was normalized to total protein content. To determine pancreatic insulin content, half of the whole pancreas was collected and placed into 5 ml Acid-Ethanol solution (1.5% HCl in 70% EtOH) overnight at −20°C. Tissue was then homogenized and incubated in the same solution overnight at −20°C. Supernatant was collected by centrifugation at 3000 x g for 10 min at 4°C, followed by neutralization with 1 M Tris pH 7.5 at 1:1 (vol/vol). Insulin and protein content of the neutralized solution were measured. Total pancreatic insulin content was normalized by total protein content.

Glucose Stimulated Insulin Secretion (GSIS) Assay

To measure GSIS in Min6 cells, cells were pre-incubated at 37°C for 2 hr in KRBH buffer (119 mM NaCl, 4.74 mM KCl, 2.54 mM CaCl2, 1.19 mM MgCl2, 1.19 mM

KH2PO4, 25 mM NaHCO3, and 10 mM HEPES, pH 7.4) containing 0.1% BSA and 0.1 mM glucose. After washing with the same buffer, cells were then incubated in the KRBH buffer containing 0.1% BSA and 0.1 mM glucose at 37°C for 30 min, followed by incubation with KRBH buffer containing 0.1% BSA and 25 mM glucose for another 30 min. The supernatant was collected for insulin measurements.

To measure GSIS in the isolated islets, islets were pre-incubated at 37°C for 2 hr in buffer containing 125 mM NaCl, 5.9 mM KCl, 2.56 mM CaCl2, 1.2 mM MgCl2, 25 mM HEPES, 0.1% BSA and 2.8 mM glucose, pH 7.4 (2.8 G buffer). Batches of around 50 islets were placed between two layers of bio-gel (Bio-Rad) in a perifusion chamber and perifused at a flow rate of 200 µL/min, at 37°C, with 2.8 G buffer for 20 min prior to the start of collecting samples. To determine the dynamic response of insulin secretion, islets were first perifused for 10 min with 2.8 G buffer, followed by 10 min with buffer containing 125 mM NaCl, 5.9 mM KCl, 2.56 mM CaCl2, 1.2 mM MgCl2, 25 mM HEPES, 0.1% BSA and 16.7 mM glucose, pH 7.4 (16.7 G buffer), prior to incubation with 2.8 G buffer for 30 min. Samples were collected every 2 min for insulin measurements. At the end of the perifusion assay, islets were lysed in RIPA buffer and total insulin content was measured. GSIS was normalized by total islet insulin content.

To measure GSIS *in vivo*, mice were starved for 6 hr and blood were collected from tail vein to determine basal insulin levels. Mice were then injected with 2 g glucose/kg of body weight, intraperitoneally. Blood samples were collected from the tail vein 15 min after glucose injection. Total blood was centrifuged at 10,000 *x g* for 10 min and the supernatant (serum) was collected for insulin measurements using Mercodia Mouse Insulin ELISA (Mercodia).

<u>Insulin and glucose tolerance tests</u>

Prior to GTT or ITT, mice were fasted for 6 h with ad libitum access to water and then administered glucose (2 g/kg) or insulin (0.75 unit/kg) intraperitoneally. Blood glucose was measured in blood samples collected from tail vein using a glucometer (Accu-Chek Performa Nano System).

<u>*In vitro* $[Ca^{2+}]_i$ imaging and data analysis</u>

In vitro imaging of whole intact islets was carried out 1-2 days after isolation from FL and KO animals. Changes in cytosolic free Ca2+ concentration were measured using fura2-AM on a fluorescence wide-field microscope (Leica DMI6000B). Single islets were attached to glass coverslips using a peptide hydrogel (Corning Inc., 354250) inside an open recording chamber (Warner Instruments), with the whole system maintained at 37°C. Prior to imaging, islets were starved for 1 h in 2.8 G buffer containing 4 uM Fura2-AM. Islets were stimulated with a glucose concentration of 16.7 mM, Fura-2 fluorescence was measured using the excitation from a xenon lamp (Sutter DG-4) and fura-2 filter set (Excitation 340/380 nm; Dichroic 415 nm; Emission 510 nm). Responses were measured as area under the curve (AUC), computed using the trapezoidal method. Due to the lack of a clear peak and drifting baseline for the FIT2 KO group, the AUC was measured for only the first 120 s of the response to high glucose. Analysis was carried out using ImageJ and Prism 7 (GraphPad).

<u>ATP/ADP Ratio</u>

FL or KO islets were pre-incubated in 2.8 G buffer for 2 hr, then incubated with a 2.8 G or a 16.7 G buffer for 15 min. ATP/ADP ratio was determined with ADP/ATP Ratio

Bioluminescence Assay Kit (Biovision), according to manufacturer's instruction.

Membrane Capacitance

Membrane capacitance were recorded from intact pancreatic islets using the standard whole cell patch clamp technique and software lock-in module of the Patchmaster software together with an EPC10/2 amplifier (HEKA Elektronik). An 800 Hz, 25 mV peak-to-peak sinusoidal wave was applied around a holding potential of –70 mV. β-cell exocytosis was triggered with 10 consecutive 500 ms depolarization pulses (-70 to 0 mV) with 100 ms intervals. β -Cells were identified based on their size (Cm > 5 pF) and discernible voltage-gated Na+ currents (6). Islets are bathed in extracellular solution containing the following: 118 mmol/L NaCl, 5.6 mmol/L KCl, 1.2 mmol/L MgCl2, 10 mmol/L CaCl2, 20 mmol/L tetraethylammonium chloride, 5 mmol/L HEPES, and 5 mmol/L glucose (310 mOsm, pH 7.4). The bath was perfused continuously, and all recordings were carried out at room temperature. Recording pipettes were pulled with P-1000 (4-7 MΩ; Sutter Instrument Co.) and filled with an internal solution containing the following: 125 mmol/L Cs-glutamate, 10 mmol/L CsCl, 10 mmol/L NaCl, 1 mmol/L MgCl2, 5 mmol/L HEPES, 0.05 mmol/L EGTA, and 3 mmol/L MgATP (300 mOsm, pH 7.3).

In situ RNA hybridization

In situ hybridization for FIT2 mRNA on pancreas tissue was performed using RNAscope 2.5 HD Detection Kit - RED (Advanced Cell Diagnostics) according to the manufacturer's instructions. Briefly, mouse pancreas was fixed with 4% PFA for 6 hr at 4 °C. The fixed tissues were then immersed gradually through a series of 10%, 20% and 30% sucrose, frozen in the Optimal Cutting Temperature (OCT) embedding media and cut at 10 μm thickness. Tissue sections were sequentially pretreated with RNAscope Hydrogen Peroxide, RNAscope Target Retrieval and RNAscope Protease Plus regeants prior to hybridization with the target oligo probes. The signals were then amplified and developed, followed by counterstaining insulin and nuclei with IHC using anti-insulin antibody, Alexa 488-labeled anti-guinea pig antibody and DAPI. Images were captured using the SP8 confocal microscope (Leica).

Ceramide analysis

Pancreatic islets were resuspended in 100 μL of butanol/methanol (1:1, v:v) spiked with 66.07 nM of C8 Ceramide (d18:1/8:0) purchased from Avanti Polar Lipids (860508). Cells were sonicated for 30 min followed by centrifugation at 14,000 g for 10 min at 22 °C. The supernatant was transferred to MS vials for analysis. The samples were analysed using an Agilent 1290 series UHPLC system connected to an Agilent 6495 QQQ mass spectrometer after separation on a ZORBAX Eclipse plus C18 column (2.1 x 50 mm, 1.8 μm, 95 Å, Agilent) at 40 °C. The injection volume was 2 μL. Solvent A consisted of 60% water/40% acetonitrile (v/v) with 10mM ammonium formate; solvent B consisted of 90% isopropanol/10% acetonitrile (v/v) with 10mM ammonium formate. The gradient started with a flow rate of 0.4 mL/min at 20%B and increased to 60% B at 2 min, 100% B at 7 min, held at 100% B until 9 min, followed by equilibration with 20% B from 9.01 min until 10.8 min. The column effluent was introduced to the Agilent 6495 QQQ mass spectrometer via AJS-ESI ion source operating under the following conditions: Gas temperature, 200°C; gas flow, 14 L/min; nebulizer, 20 psi; sheath gas temperature, 250°C; sheath gas flow, 11 L/min; capillary, 3500 V. Mass spectrometry analysis was performed in positive ion

mode with dynamic scheduled multiple reaction monitoring (dMRM). Mass spectrometry settings, LC-MS gradient and MRM transitions for each lipid class were adapted from a previously published method (53). Data analysis was performed on Agilent MassHunter Quantitative analysis software. Relative quantitation was based on one-point calibration with Ceramide d18:1/8:0. The data were further normalized to the total number of islets in each sample.

Co-immunoprecipitation assay

MIN6 cells, transiently co-transfected with pcDNA3.1-FIT2/V5-His and GFP-tagged MARCH6 (BC059190) Mouse Tagged ORF Clone (Origene), were pre-treated with 10 μM MG132 (Cell Signaling Technology) for 2 h and subsequently treated with 300 μM palmitate (BSA-conjugated) for 6 h. Cells were then lysed in IP lysis buffer (ThermoFisher Scientific), supplemented with protease inhibitor cocktail. Co-immunoprecipitation assay was then performed on the protein lysates using Pierce™ Classic IP Kit (ThermoFisher Scientific) with either Anti-6X His tag antibody (Abcam) or Rabbit IgG (Cell Signaling Technology) in accordance with the manufacturer's instructions. The eluted fraction was then subjected to immunoblotting assay and MARCH6 was detected using Mouse monoclonal turboGFP antibody (Origene).

S-Palmitoylation Assays

Thiol side-chain protection assay: MIN6 cells, transiently transfected with pcDNA3.1-FIT2/V5-His, were pre-treated with 10 μM MG132 (Cell Signaling Technology) for 2 h and thereafter treated with 300 μM palmitate (BSA-conjugated) for 6 h. S-palmitoylation assay was then performed using CAPTUREome™ S-Palmitoylated Protein Kit (Badrilla) in accordance with manufacturer's instruction. Briefly, cell lysis and free thiol blocking (blocking of free thiols on non-palmitoylated cysteine residue) was performed by incubating cells with the provided lysis buffer for 4 h with constant shaking. Proteins were then precipitated using acetone, and subsequently resuspended in the provided binding buffer. 30 μl of resuspended proteins were collected as the Input Fraction (IF), while the remainder was split into two tubes: "experimental" and "negative control". In the experimental tube, proteins were treated with thioester cleavage reagent, which cleaves off acyl groups from the protein, resulting in the exposure of a free thiol group. Treated samples were then subjected to CAPTUREome™ resin for 2.5 h at room temperature, where proteins with free thiols were captured by the resin. 50 μl of flowthroughs were collected as the cleaved unbound fraction (cUF). S-acylated proteins, which were captured by the CAPTUREome™ resin, were eluted by incubating the resin in 50 μl of 2x laemmli buffer at 60°C for 10 min (cleaved Bound Fraction (cBF)). In the negative control tube, proteins were treated with acyl-preservation reagent, which preserves acyl groups on the protein. Treated samples were then subjected to CAPTUREome™ resin for 2.5 h at room temperature. 50 μl of flowthroughs were collected as preserved unbound fraction (pUF). Proteins were eluted from the CAPTUREome™ resin by incubation of resin with 50 μl of 2x laemmli buffer at 60°C for 10 min (preserved Bound Fraction (pBF)).

Click-chemistry S-palmitoylation Assay: MIN6 cells, transiently transfected with pcDNA3.1-FIT2/V5-His, were pre-treated with 10 μM MG132 (Cell Signaling Technology) for 2 h and thereafter treated with 200 μM Click-IT™ Palmitic Acid, Azide (ThermoFisher Scientific) for 6 h. Cells were then lysed in 50 mM Tris-HCL, pH 8.0, supplemented with 1% SDS and protease inhibitor cocktail. Palmitoylated proteins were conjugated using the Click-iT® Protein Reaction Buffer Kit

(ThermoFisher Scientific), in accordance with manufacturer's instructions, without modifications.

SUPPLEMENTARY TABLE OF REAGENTS

REAGENT or RESOURCE	SOURCE	IDENTIFIER	Lot
**Antibodies**			
Rabbit polyclonal anti-FIT2	Dr. David L. Silver	(7)	
Rabbit polyclonal anti-Seipin	Abcam	Cat# ab106793	
Rabbit polyclonal anti-Perilipin-2	Novus Biologicals	Cat#NB110-40877	Lot# F-1
Rabbit polyclonal anti-CHOP (F-168)	Santa Cruz Biotechnology	Cat# sc-575	Lot# F2613
Rabbit polyclonal anti-ATF4 (C-20)	Santa Cruz Biotechnology	Cat# sc-200	Lot# G0115
Rabbit polyclonal anti-p-eIF2α (Ser 52)	Santa Cruz Biotechnology	Cat# sc-101670	Lot# C0714
Rabbit monoclonal anti-IRE1α (14C10)	Cell Signaling Technology	Cat#3294	Lot# 9
Rabbit polyclonal anti-p-IRE1α (p Ser724)	Novus Biologicals	Cat#NB100-2323	Lot# AF-3
Mouse monoclonal anti-β-actin (AC-15)	Abcam	Cat#6276	Lot# GRC3207325-9
Rabbit polyclonal anti-6XHis	Abcam	Cat#ab9108	Lot# GR3246563-1
Mouse monoclonal turboGFP antibody, clone OTI2H8	Origene	Cat#TA150041	Lot#W004
Normal Rabbit IgG	Cell Signaling Technology	Cat#2729	Lot#8
Horse anti-mouse IgG (HRP-linked)	Cell Signaling Technology	Cat#7076	Lot# 31
Goat anti-rabbit IgG (HRP-linked)	Cell Signaling Technology	Cat#7074	Lot# 28
Guinea pig polyclonal anti-Insulin	Dako	Cat#A0564	Lot# 10088287
Mouse monoclonal anti-glucagon (K79bB10)	Sigma-Aldrich	Cat#G2654	
Monoclonal Anti-Ceramide antibody produced in mouse	Sigma-Aldrich	Cat#C8104	Lot# SLCC3937
Rat monoclonal anti-somatostatin (YC7)	Merk Millipore	Cat#MAB354	
Goat anti-guinea pig IgG, Alexa Fluor 488	Thermo Fisher Scientific	Cat#A11073	Lot# 1737010
Goat anti-guinea pig IgG, Alexa Fluor 568	Thermo Fisher Scientific	Cat#A11075	Lot# 1692965
Goat anti-mouse IgG, Alexa Fluor 488	Thermo Fisher Scientific	Cat#A28175	Lot# 1611153
Goat anti-Mouse IgM (Heavy chain) Cross-Adsorbed Secondary Antibody, Alexa Fluor 488	Thermo Fisher Scientific	Cat#A21042	Lot# 2079371
Goat anti-rat IgG, Alexa Fluor 647	Thermo Fisher Scientific	Cat#A21247	
Goat anti-rabbit IgG, Alexa Fluor 488	Thermo Fisher Scientific	Cat#A32731	Lot# 1622775
**Bacterial and Virus Strains**			
FIT2 MISSION shRNA lentiviral transduction particles	Sigma-Aldrich	Cat#SHCLNV-NM_173397 (TRCN0000125380)	
**MISSION pLKO.1-puro non-target shRNA control transduction particles**	Sigma-Aldrich	Cat#SHC016V	
**Chemicals, Peptides, and Recombinant Proteins**			
BODIPY 493/503	Thermo Fisher Scientific	Cat#D3922	

Fura-2, AM	Thermo Fisher Scientific	Cat#F1225	
DAPI	Thermo Fisher Scientific	Cat#D1306	
Propidium iodide	Thermo Fisher Scientific	Cat#P1304MP	
Palmitic acid	Sigma-Aldrich	Cat#57-10-3	
Click-IT™ Palmitic Acid, Azide (15-Azidopentadecanoic Acid)	Thermo Fisher Scientific	Cat#C10265	
Biotin Alkyne (PEG4 carboxamide-Propargyl Biotin)	Thermo Fisher Scientific	Cat#B10185	
Oleic acid	Sigma-Aldrich	Cat#112-80-1	
Stearic acid	Sigma-Aldrich	Cat#57-11-4	
Palmitoleic acid	Sigma-Aldrich	Cat#P9417	
Sodium hydroxide	Sigma-Aldrich	Cat#1310-73-2	
D-(+)-Glucose	Sigma-Aldrich	Cat#50-99-7	
Humalog U-100 insulin	Eli Lilly	Cat#HP8799	
MG-132	Cell Signaling Technology	Cat#2194	
2-Bromopalmitic acid	Sigma-Aldrich	Cat#21604	
Eeyarestatin	Sigma-Aldrich	Cat#E1286	
Cerulenin	Sigma-Aldrich	Cat#C2389	
**Critical Commercial Assays**			
RNAscope 2.5 HD detection kit (red)	Advanced Cell Diagnostics	Cat#322360	
Pierce BCA protein assay kit	Thermo Fisher Scientific	Cat#23225	
Lipofectamine 2000 transfection reagent	Thermo Fisher Scientific	Cat#11668019	
Fast SYBR green master mix	Thermo Fisher Scientific	Cat#4385617	
Pierce™ Classic IP Kit	Thermo Fisher Scientific	Cat#26146	
Pierce® IP Lysis Buffer	Thermo Fisher Scientific	Cat# 87788	
RIPA buffer	Sigma-Aldrich	Cat#R0278	
Protease inhibitor cocktail	Sigma-Aldrich	Cat#P8340	
SignalFire ECL reagent	Cell Signaling Technology	Cat#6883	
ADP/ATP ratio bioluminescence assay kit	BioVision	Cat#255-200	
NucleoSpin RNA XS	Macherey-Nagel	Cat#740902	
NucleoSpin RNA	Macherey-Nagel	Cat#740955	
Mouse Insulin ELISA	Mercodia	Cat#10-1247-01	
Ultrasensitive mouse Insulin ELISA	Mercodia	Cat#10-1249-01	
Apo-ONE homogeneous caspase-3/7 assay	Promega	Cat#G7790	
CAPTUREome™ S-Palmitoylated Protein Kit	Badrilla	Cat#K010-311	
**Deposited Data**			
RNAseq Data	GEO	GEO: GSE133939	
**Experimental Models: Cell Lines**			
Min6 cells	Dr. Jun-ichi Miyazaki	(1)	

Min6 cells stably expressing control shRNA	This study	N/A	
Min6 cells stably expressing FIT2 shRNA	This study	N/A	
Human: HEK 293T/17	ATCC	Cat#CRL-11268	
**Experimental Models: Organisms/Strains**			
Mouse: C57BL/6J	The Jackson Laboratory	Cat#000664	
Mouse: FIT2fl/fl	Dr. David L. Silver	(8)	
Mouse: B6.Cg-Tg (Ins2-cre) 25Mgn/J	The Jackson Laboratory	Cat#003573	
Mouse: 129S1/SvImJ	The Jackson Laboratory	Cat#002448	
Mouse: B6.Cg-Tg (Ins2-cre) 25Mgn/J:129S1/SvImJ	This study	N/A	
Mouse: C57BL/6J:129S1/SvImJ	This study	N/A	
Mouse: B6.Cg-Tg(Ins2-cre)25Mgn/J:FIT2fl/fl	This study	N/A	
Mouse: B6.BKS (D)-Lepr^{db}/J	The Jackson Laboratory	Cat#000697	
**Oligonucleotides**			
ON-TARGETplus March6 siRNA SmartPool	Horizon Discovery	Cat# L-055065-01-0020	
ON-TARGETplus Amfr siRNA SmartPool	Horizon Discovery	Cat#L-048676-01-0020	
See Supplemental Table 2 for primer sequences	This study	N/A	
**Recombinant DNA**			
pcDNA3.1 mammalian expression vector	Thermo Fisher Scientific	Cat#V79020	
pcDNA3.1-FIT2	Dr. David L. Silver	(9)	
pcDNA3.1-FIT2/V5-His	Dr. David L. Silver	(9)	
pcDNA3.1-FIT2(C7A)/V5-His	Bio Basic Inc.	N/A	
pcDNA3.1-FIT2(C70A)/V5-His	Bio Basic Inc.	N/A	
pcDNA3.1-FIT2(C140A)/V5-His	Bio Basic Inc.	N/A	
pcDNA3.1-FIT2(C251A)/V5-His	Bio Basic Inc.	N/A	
pcDNA3.1-FIT2(C7/70/140/251A)/V5-His	Bio Basic Inc.	N/A	
March6 (BC059190) Mouse Tagged ORF Clone	Origene	CAT#MG207831	
**Software and Algorithms**			
GraphPad Prism 7	GraphPad	https://www.graphpad.com	
Image Lab 5.0	Bio-Rad	https://www.bio-rad.com/en-sg/product/image-lab-software	
Leica LAS X software	Leica	https://www.leica-microsystems.com/products/microscope-software	
NIS-Elements software	Nikon	https://www.microscope.healthcare.nikon.com/products/software	
ImageJ	N/A	https://imagej.net/Welcome	

Other			
Chow diet	Altromin	Cat#1324	
Western diet	Research Diets	Cat#D12079B	

SUPPLEMENTARY INFORMATION REFERENCES

1. Miyazaki J, Araki K, Yamato E, Ikegami H, Asano T, Shibasaki Y, et al. Establishment of a pancreatic beta cell line that retains glucose-inducible insulin secretion: special reference to expression of glucose transporter isoforms. Endocrinology. 1990;127(1):126-32.
2. Dobin A, Davis CA, Schlesinger F, Drenkow J, Zaleski C, Jha S, et al. STAR: ultrafast universal RNA-seq aligner. Bioinformatics (Oxford, England). 2013;29(1):15-21.
3. Li B, Dewey CN. RSEM: accurate transcript quantification from RNA-Seq data with or without a reference genome. BMC Bioinformatics. 2011;12(1):323.
4. Trapnell C, Hendrickson DG, Sauvageau M, Goff L, Rinn JL, Pachter L. Differential analysis of gene regulation at transcript resolution with RNA-seq. Nature biotechnology. 2013;31(1):46-53.
5. Trapnell C, Williams BA, Pertea G, Mortazavi A, Kwan G, van Baren MJ, et al. Transcript assembly and quantification by RNA-Seq reveals unannotated transcripts and isoform switching during cell differentiation. Nature biotechnology. 2010;28(5):511-5.
6. Hiriart M, Matteson DR. Na channels and two types of Ca channels in rat pancreatic B cells identified with the reverse hemolytic plaque assay. J Gen Physiol. 1988;91(5):617-39.
7. Kadereit B, Kumar P, Wang WJ, Miranda D, Snapp EL, Severina N, et al. Evolutionarily conserved gene family important for fat storage. Proceedings of the National Academy of Sciences of the United States of America. 2008;105(1):94-9.
8. Miranda DA, Kim JH, Nguyen LN, Cheng W, Tan BC, Goh VJ, et al. Fat Storage-inducing Transmembrane Protein 2 Is Required for Normal Fat Storage in Adipose Tissue. The Journal of biological chemistry. 2014;289(14):9560-72.
9. Gross DA, Zhan C, Silver DL. Direct binding of triglyceride to fat storage-inducing transmembrane proteins 1 and 2 is important for lipid droplet formation. Proceedings of the National Academy of Sciences of the United States of America. 2011;108(49):19581-6.

Data Table S1. Oligonucleotides

Name	Forward primer (5´- 3´)	Reverse primer (5´- 3´)
**qPCR**		
Fit2	CACGACTTGACCCAGAAAGTG	GGAGGAAGTCCTGGAGAGAAA
Ins2	CCATCAGCAAGCAGGAAGCCTATC	CCCCACACACCAGGTAGAGAGCC
MafA	GAGGTCATCCGACTGAAAC	CACTTCTCGCTCTCCAGA
Pdx-1	GAAATCCACCAAAGCTCAC	AAGAATTCCTTCTCCAGCTC
NeuroD1	AACAACAGGAAGTGGAAAC	TTTCTTGTCTGCCTCGTG
β-actin	GACAGGATGCAGAAGGAGAT	TTGCTGATCCACATCTGCTG
**Genotyping**		
Cre5	GCAAGTTGAATAACCGGAAATGGTT	AGGGTGTTATAAGCAATCCCCAGAA
FL146	CCCATGTTAGTAGAAGCAAGCGTGA	ACAAGGAAAGCCTCGTTAAAACCAA
